# The m^6^A writer RBM15 drives the growth of triple-negative breast cancer cells through the stimulation of serine and glycine metabolism

**DOI:** 10.1038/s12276-024-01235-w

**Published:** 2024-06-03

**Authors:** Su Hwan Park, Jin-Sung Ju, Hyunmin Woo, Hye Jin Yun, Su Bin Lee, Seok-Ho Kim, Balázs Győrffy, Eun-jeong Kim, Ho Kim, Hee Dong Han, Seong-il Eyun, Jong-Ho Lee, Yun-Yong Park

**Affiliations:** 1 Department of Health Sciences, The Graduate School of Dong-A University, Busan, Republic of Korea; 2 Asan Institute for Life Sciences, Asan Medical Center, Seoul, Republic of Korea; 3 Department of Life Science, Chung-Ang University, Seoul, Republic of Korea; 4 Department of Medicinal Biotechnology, College of Health Science, Dong-A University, Busan, Republic of Korea; 5 Department of Bioinformatics, Semmelweis University, H-1094 Budapest, Hungary; 6 Department of Biophysics, Medical School, University of Pecs, H-7624 Pecs, Hungary; 7 Cancer Biomarker Research Group, Institute of Molecular Life Sciences, Research Centre for Natural Sciences, H-1117 Budapest, Hungary; 8 Division of Life Science and Chemistry, College of Natural Science, Daejin University, Pocheon, Republic of Korea; 9 Department of Immunology, School of Medicine, Konkuk University, Chungcheongbuk-Do, Republic of Korea

**Keywords:** Breast cancer, Translational research

## Abstract

*N*^6^-adenosine methylation (m^6^A) is critical for controlling cancer cell growth and tumorigenesis. However, the function and detailed mechanism of how m^6^A methyltransferases modulate m^6^A levels on specific targets remain unknown. In the current study, we identified significantly elevated levels of RBM15, an m^6^A writer, in basal-like breast cancer (BC) patients compared to nonbasal-like BC patients and linked this increase to worse clinical outcomes. Gene expression profiling revealed correlations between RBM15 and serine and glycine metabolic genes, including PHGDH, PSAT1, PSPH, and SHMT2. RBM15 influences m^6^A levels and, specifically, the m^6^A levels of serine and glycine metabolic genes via direct binding to target RNA. The effects of RBM15 on cell growth were largely dependent on serine and glycine metabolism. Thus, RBM15 coordinates cancer cell growth through altered serine and glycine metabolism, suggesting that RBM15 is a new therapeutic target in BC.

## Targeting RBM15: a novel therapeutic approach for breast cancer

RNA methylation, a key process controlling gene activity, involves proteins like RNA-binding motif protein 15 (RBM15). Its role in breast cancer is unclear. Studies show RBM15 is highly active in a type of breast cancer called triple-negative and is linked to patient outcomes. It controls genes related to serine and glycine metabolism; substances that help cancer cells grow. The research demonstrates that RBM15 controls genes involved in the cancer metabolism of serine and glycine, two types of amino acids, which contributes to cancer cell proliferation. This suggests that targeting RBM15 can be new therapeutic approaches for triple-negative breast cancer patients.

This summary was initially drafted using artificial intelligence, then revised and fact-checked by the author.

## Introduction

RNA methylation is a pivotal molecular process that precisely regulates gene expression by regulating protein translation at the post-transcriptional stage^1^. RNA methylation, including *N*^6^-adenosine methylation (m^6^A), is the predominant RNA modification^2,3^. Recently developed RNA sequencing technologies enable the assessment of RNA modification sites, including RNA methylation sites^3^.

The three classes of m^6^A methyltransferases, known as writers, readers, and erasers, play crucial roles in human cancers^4,5^. Writers, such as methyltransferase-like (METTL) 3 and 14, contribute to cancer cell growth and patient prognosis^5^. Readers, including YT521-B homology (YTH) domain-containing proteins (YTH domain family [YTHDF] and YTH domain-containing 1 and 2), heterogeneous nuclear ribonucleoprotein proteins, and insulin-like growth factor-2 mRNA-binding proteins (IGF2BP1-3), also participate in cancer cell growth^6^. In contrast, erasers, such as fat mass and obesity-associated protein (FTO), have demethylase activity and function as tumor suppressors in cancers^2^.

m^6^A methyltransferases function as master regulators of tumorigenesis and can frequently lead to drug resistance, resulting in a worsened patient prognosis^7^. Recently, a METTL3-selective catalytic inhibitor was developed and found to be effective in treating leukemia^8^, suggesting that m^6^A methyltransferases are potential targets for treating multiple human cancer types. Although m^6^A methyltransferases are clearly involved in tumorigenesis and influence the clinical outcomes of patients, including chemosensitivity, their gene regulatory mechanisms are not fully understood. Moreover, how m^6^A methyltransferases regulate their target gene expression remains unknown, and the genes affected by specific m^6^A methyltransferases are still under investigation.

Recently, serine and glycine biosynthesis has emerged as a new therapeutic target for cancer. Serine and glycine metabolism is highly activated in many human cancers and plays crucial roles in cancer cell proliferation, leading to tumor growth^9^. Thus, serine and glycine contribute to cellular biosynthesis and homeostasis in rapidly growing cancer cells. Both serine and glycine in the extracellular environment can be taken up into cells via various transporters. In addition, serine can be synthesized via the serine synthesis pathway (SSP)^9,10^. In the SSP, phosphoglycerate dehydrogenase (PHGDH) catalyzes the first step of the NAD^+^-dependent oxidation of 3-phosphoglycerate 3-PG to 3-phosphohydroxypyruvate (3PHP); subsequently, phosphoserine aminotransferase 1 (PSAT1) converts 3PHP to 3-phosphoserine (3-PS); and finally, serine is generated from phosphoserine through phosphoserine phosphatase (PSPH)-mediated dephosphorylation^9–11^. The (synthesized or taken up) intracellular serine can be directly converted by a hydroxymethyltransferase (SHMT) 1/2 reaction^9,10^. Recent investigations have demonstrated that blocking serine biosynthesis significantly inhibits the growth of cancer cells, indicating that rapidly proliferating cells are dependent on serine^12^.

In this study, we found that the expression of the RNA-binding motif protein 15 (RBM15) m^6^A writer was significantly upregulated in triple-negative breast cancer (TNBC) cells and was closely associated with clinical outcomes. RBM15 exhibited oncogenic effects by regulating serine and glycine metabolism-related genes, such as PHGDH, PSAT1, PSPH, and SHMT2, leading to increased serine and glycine flux. Similar to those of RBM15, higher expression levels of these genes were associated with inferior patient outcomes. Our study revealed that RBM15 could unexpectedly regulate serine and glycine metabolism in breast cancer (BC) cells. We propose that RBM15 targeting can be beneficial for BC patients.

## Materials and methods

### Data processing

The gene expression datasets were downloaded from the Gene Expression Omnibus (GEO) (GSE76275, GSE21653, GSE65216, GSE11121, GSE22358, GSE25066, GSE106977, GSE73893, GSE31519, GSE58812, GSE76250, GSE22226, GSE16446, GSE3494, GSE32646, GSE41998, GSE22093, GSE21094, GSE34138, and GSE31448) and the TCGA portal (https://www.cbioportal.org). All the data were normalized using the quantile normalization method in the R program before being used for the analysis.

### Survival analysis

Published patient data were used for survival and receiver operating characteristic (ROC) curve analyses. The association of RBM15 and target gene expression (rank ordered and split into high or low) with patient survival was assessed using the log-rank test. The results were visualized using Kaplan‒Meier plots.

### Cell lines and culture

MDA-MB-231 cells (#30026, Korean Cell Line Bank) and Hs 578 T cells (#30126, Korean Cell Line Bank, Republic of Korea) were maintained in RPMI 1640 and DMEM (#LM011, LM001, Welgene, Republic of Korea) supplemented with 10% fetal bovine serum and 1% antibiotic–antimycotic solution (#LS 203-01, Welgene). All the cell lines were grown at 37 °C in the presence of 5% CO_2_. For serine and glycine deprivation experiments, the cells were cultured in serine- and glycine-free DMEM supplemented with 10% dialyzed fetal bovine serum (#26400044, Thermo Fisher, Waltham, MA) and 1% antibiotics.

### Immunoblotting

Cell lysates were prepared using RIPA buffer (#IBS-BR002, Intron Biotechnology, Republic of Korea) containing Halt^TM^ Protease & Phosphatase Inhibitor Cocktail (#78442, Thermo Scientific). Protein lysates were separated on 8%, 10%, and 12% SDS–PAGE gels and then transferred to NC or PVDF membranes. Protein detection was performed using anti-RBM15 (#60386, Cell Signaling, Danvers, MA; #66059, Proteintech, Rosemont, IL), anti-SHMT2 (#33443, Cell Signaling), anti-PHGDH (#66350, Cell Signaling), anti-PSAT1 (#67619, Proteintech), anti-PSPH (#13503-R001, Sino Biological), anti-β-actin (#4967, Cell Signaling), anti-HK2 (#sc-130858, Santa Cruz, Dallas, TX), anti-PFKP (#12746, Cell Signaling), anti-PFKM (#sc-67028, Santa Cruz), anti-PFKL (#sc-393713, Santa Cruz), anti-PFK2 (#sc-377416, Santa Cruz), anti-aldolase (#sc-390733, Santa Cruz), anti-GAPDH (#sc-47724, Santa Cruz), anti-PGK1 (#68540, Cell Signaling), anti-PGAM1 (#GTX629745, GeneTex, Irvine, CA), anti-enolase (#sc-271384, Santa Cruz), anti-PKM2 (#3198, Cell Signaling), anti-LDHA (#sc-137243, Santa Cruz), anti-Vinculin (#13901, Cell Signaling), anti-β-tubulin (#T4026, Sigma Aldrich, St. Louis, MO), and anti-FLAG (#F3165, Sigma Aldrich). The band intensity was quantified by using ImageJ 1.53e software (National Institutes of Health, Bethesda, MD).

### RNA-Immunoprecipitation (RNA-IP)

RNA-IP was performed using an Active Motif Magnetic RNA ChIP Kit (#53024, Active Motif, Carlsbad, CA) according to the manufacturer’s instructions. BC cells were cultured to 80–90% confluence in 10 cm plates. The cells were crosslinked with 1% formaldehyde and washed with 1× PBS. The cells were then lysed in lysis buffer containing a protease inhibitor and an RNase inhibitor. Crosslinked nuclear lysates were subjected to six rounds of sonication on ice. Each set consisted of 20 s of sonication with 30 s of rest on ice between sets. The digested chromatin was immunoprecipitated overnight with primary RBM15 antibody at 4 °C. Normal mouse anti-IgG was used in parallel as a control. Then, pulldown with magnetic beads and crosslinking at 65 °C for 1.5 h were performed. RNA purification was then performed using the easy-BLUE^TM^ Total RNA Extraction Kit (#17061, Intron Biotechnology) according to the manufacturer’s instructions. The amount of immunoprecipitated RNA was quantified using target gene primers.

### Lentivirus generation by short hairpin RNA (shRNA) transfection and infection of BC cells

pLKO.1 shRBM15 (TRCN0000074704, TRCN0000074705, and TRCN0000074707), shMETTL3 (TRCN0000058391), shMETTL14 (TRCN0000015933), shIGF2BP1 (TRCN0000218079), shIGF2BP2 (TRCN0000255463), and shIGF2BP3 (TRCN0000074675) were purchased from Sigma-Aldrich. Lentiviruses were generated, followed by transduction of the lentiviral vectors psPAX2 and pMD2.G into Lenti-X 293 T cells using Lipofectamine® 3000 Reagent (#L3000015, Invitrogen, Waltham, MA). Lentivirus-containing supernatants were collected 48 h after transduction. BC cell lines were infected with polybrene (8 µg/ml, #TR-1003-G, Sigma Aldrich) and selected with puromycin for 3 days.

### DNA construction

Human WT-RBM15 or the ΔRRM-RBM15 mutant was cloned and inserted into the pCDH-CMV-EF1-Puro plasmid (#CD510B-1, System Biosciences, Palo Alto, CA). The ΔSPOC-RBM15 mutant plasmid was kindly provided by Dr. Slade (Medical University of Vienna, Austria)^13^, and the WT or ΔSPOC-RBM15 mutant was subcloned and inserted into the pcDNA3.1/Hygro (+) plasmid (#V87020, Thermo Fisher Scientific).

### siRNA transfection

siRNAs (SASI_Hs01_00219652: siRBM15 #1, SASI_Hs01_00219654: siRBM15 #2) were purchased from Sigma-Aldrich and transfected into BC cell lines according to the manufacturer’s protocol (Lipofectamine® 3000 Reagent, #L3000015, Invitrogen). After transfection, the cells were used for the indicated assays.

### Quantitative PCR

Total RNA was extracted using the easy-BLUE^TM^ Total RNA Extraction Kit (Intron Biotechnology) according to the manufacturer’s instructions. Quantitative PCR was performed using gene-specific IDT primers (IDT, Inc., Coralville, IA) and the SensiFAST^™^ Probe Hi-ROX One-Step Kit (#BIO-77005, Bioline, UK) or purchased from Bionics (oligo synthesis service, Republic of Korea) for gene expression analysis. Each gene was normalized against the human PPIA gene. The following primers were purchased from IDT: PPIA (Hs.PT.58 v.38887593.g), RBM15 (Hs.PT.58.2989627), ACTB (Hs.PT.39a.22214847), SHMT2 (Hs.PT.58.1292846), PSAT1 (Hs.PT.58.20540177), PSPH (Hs.PT.58.39334783), and PHGDH (Hs.PT.58.2437570). Bionics primers were as follows: METTL3 (For: 5′-TTGTCTCCAACCTTCCGTAGT-3′, Rev: 5′-CCAGATCAGAGAGGTGGTGTAG-3′), METTL14 (For: 5′-TGACATCAGAGAACTAACACCCA-3′, Rev: 5′- GATCGAGGTGCTGCAATCTC-3′), IGF2BP1 (For: 5′-GACCCCTGATGAGAACGAC-3′, Rev: 5′-TGGTTACTCTGTCCCTTCTGA-3′), IGF2BP2 (For: 5′-ATCGTCAGAATTATCGGGCA-3′, Rev: 5′-GCGTTTGGTCTCATTCTGTC-3′), and IGF2BP3 (For: 5′-AGACACCTGATGAGAATGACC-3′, Rev: 5′-GTTTCCTGAGCCTTTACTTCC-3′).

### Cell viability assay

BC cells (3 × 10^3^) were plated in triplicate in 96-well plates and incubated for 96 h. Cell viability was measured using CCK-8 (#CK04, Dojindo, Japan) or WST-8 (#QM2500, Biomax, Republic of Korea) assays following the manufacturer’s protocol. The assay reagent was added to the plates and incubated at 37 °C for 1 h. The absorbance was then measured at 450 nm.

### Colony formation assay

For the colony formation assay, MDA-MB-231 and Hs 578 T cells (1 × 10^3^) were seeded in six-well plates at 37 °C. The cells were cultured for 14 days. The cells were then washed with 1× PBS and fixed with methanol for 20 min. After fixation, the cells were stained with 0.05% crystal violet for 1 h and washed with dH_2_O. The colonies were then counted manually.

### Cell cycle analysis

For cell cycle analysis, cells were cultured to 80% confluence in 10 cm plates. The cells were washed with 1× PBS and trypsinized, after which 1× PBS was added to the cells. The cells were then fixed with 75% EtOH and stained with propidium iodide (PI) (Sigma-Aldrich) followed by the addition of RNase A at 4 °C for 1 h in the dark. PI-stained cells were analyzed using a CytoFLEX flow cytometer (Beckman Coulter, Brea CA). Flow cytometry data were analyzed using CytExpert software (Beckman Coulter, Brea, CA).

### Gene expression profiling (microarray analysis)

Microarray analysis was performed as described previously^14^. Total RNA was isolated from the indicated cell lines using the mirVana RNA Isolation Kit (#AM1560, Ambion, Inc., Austin, TX). In brief, 1 µg of total RNA was used for labeling and hybridization according to the manufacturer’s instructions (#AMIL1791, Ambion, Inc.). The labeled samples were processed with a bead chip, which was washed and scanned using an Illumina BeadArray Reader (Illumina, Inc., San Diego, CA). The data were normalized using the quantile normalization method in the Linear Models for Microarray Data (LIMMA) package in the R program. The data were deposited in GEO (GSE183314).

### RNA m^6^A quantification

The m^6^A level in 600 ng of RNA extracted from the indicated BC cell lines was measured using the EpiQuik^TM^ m^6^A RNA Methylation Quantification Kit (#P-9005, Epigentek, Farmingdale, NY) according to the manufacturer’s instructions.

### MeRIP-qPCR

MeRIP was performed using a CUT&RUN m^6^A RNA Enrichment (MeRIP) Kit (#P-9018-24, EpiQuik, Farmingdale, NY) according to the manufacturer’s instructions. In brief, 100–150 μg of RNA was isolated from BC cells and incubated with an anti-m^6^A monoclonal antibody for 2 h at 4 °C. The mixture was then incubated with RNA beads, and RNA was extracted using the easy-BLUE^TM^ Total RNA Extraction Kit (#17061, Intron Biotechnology) according to the manufacturer’s instructions. The purified RNA was used for qRT‒PCR analysis with the indicated probes.

### IHC analysis and scoring

IHC analysis of the TMA was performed using paraffin-embedded tissue sections purchased from BioCoreUSA (#B-120Bre-1, Philadelphia, PA). The expression of RBM15 (#66059-1-Ig, Proteintech) was assessed using the VECTASTAIN Elite ABC Kit (#PK-6200, Vector Laboratories, Newark, CA). The tissue sections were then incubated with 3,3′-diaminobenzidine (#SK-4100, Vector Laboratories, Newark, CA), and the nuclei were stained with hematoxylin. Six randomly chosen fields per slide were analyzed, and the results were averaged. The tissue sections were quantitatively scored according to the percentage of positive cells and staining intensity, as defined previously^15^. The following proportion scores were assigned: 0 if 0% of the tumor cells showed positive staining, 0.1–1.0 if 0.1%–1% of the cells were stained, 1.1–2.0 if 1.1%–10% were stained, 2.1–3.0 if 11%–30% were stained, 3.1–4.0 if 31%–70% were stained, and 4.1–5.0 if 71%–100% were stained. The staining intensity was rated on a scale of 0–3 (0, negative; 1, weak; 2, moderate; and 3, strong). The proportion and intensity scores were then combined to obtain a total score (range, 0–8), as described previously^15^.

### Computational analysis of sequencing data

A stringent quality filter process was applied using FastQC and Trim Galore (http://www.bioinformatics.babraham.ac.uk). RIP-seq data were mapped to the human genome (GRCh38/hg38), and irCLIP-seq data were mapped to the mouse genome (GRCm38/mm10) using STAR^16^. The mapped BAM files were converted to reads per genome coverage (RPGC)-normalized BigWig file using bamCoverage in deepTools (ver. 3.5.1)^17^. Peak calling of irCLIP-seq in GSE154709 was performed using PureCLIP (ver. 1.3.1)^18^. Motif enrichment analysis at the binding site of RIP-seq and CLIP-seq was performed using HOMER^19^. The lengths of the motifs in HOMER were set to six and eight, and the other parameters were set at default values. The motif locations and m^6^A sites in the GTF file were visualized using Integrated Genome Viewer (IGV; ver. 2.9.2, http://software.broadinstitute.org/software/igv).

### Analysis of publicly available RIP-seq and CLIP-seq data

For identification of the RBM15-binding sites in PHGDH, PSAT1, PSPH, and SHMT2 in humans and mice, RIP-seq data using an RBM15 antibody were downloaded from GSE73893^20^, and irCLIP-seq data in which RBM15 and RNA were crosslinked were downloaded from GSE154709^21^. For analysis of the m^6^A peaks in PHGDH, PSAT1, PSPH, and SHMT2 in humans, miCLIP data (GSE71154^22^, GSE98623^23^, GSE122948^24^, GSE63753^25^, and GSE86336^26^) were also analyzed.

### Animal experiment

All animal procedures and maintenance conditions were approved by the Dong-A University Institutional Animal Care and Use Committee (DIACUC-21-47). For xenograft experiments, 1 × 10^6^ MDA-MB-231 cells stably expressing shGFP or shRBM15 were subcutaneously injected into 4-week-old female nude mice (OrientBio, Republic of Korea). The mice were sacrificed at 40 days (*n* = 10 mice per group). The tumor volume was measured using calipers every 5 days based on the following formula: tumor volume (mm^3^) = length × (width)^2^/2. The tumor weights were also recorded.

### Antitumor efficacy of CH-NP-RBM15 siRNA

For induction of tumorigenesis, MDA-MB-231 cells (1 × 10^6^) were subcutaneously injected into the mice (4-week-old female nude mice; OrientBio). CH-NP-control siRNA (5′-UUCUCCGAACGUGUCACGU[dT][dT]-3′) or CH-NP-RBM15 siRNA (5′-CUGUAACGGAGAGUGAUUU [dT][dT]-3′) was administered twice a week via intravenous injection at a dose of 5 μg of siRNA per mouse (*n* = 10 mice per group). The treatments were continued until the control group became moribund (typically at 4–5 weeks), at which point all the mice were sacrificed. The tumor volume was measured as described above.

### Measurements of serine and glycine metabolite levels

The intracellular serine and glycine levels were measured using a serine assay kit (#K743, BioVision, Exton, PA) and glycine assay kit (#K589, BioVision), respectively, according to the manufacturer’s instructions.

## Results

### RBM15 is overexpressed and associated with clinical outcomes in BC patients

**Fig. 1:**
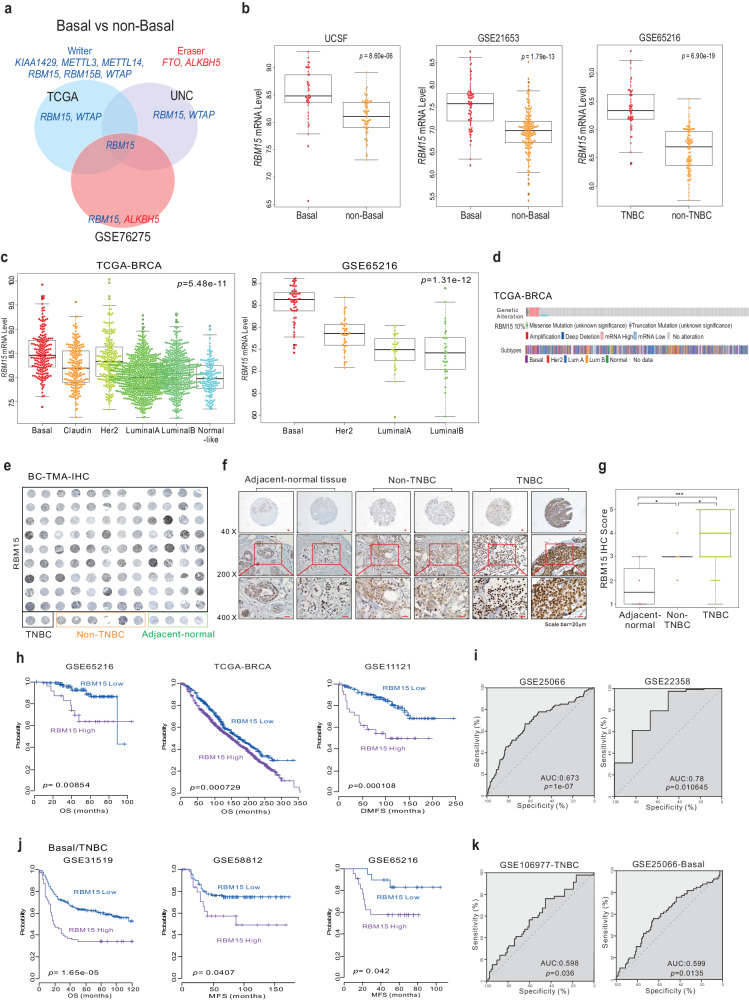
RBM15 expression and clinical outcomes in BC patients. **a** Venn diagram of genes showing significant differential expression between basal and non-basal BC tissues in three independent BC patient cohorts. A univariate test using class comparison analysis in the BRB array tool was performed. **b** RBM15 mRNA expression (log_2_) levels in basal/TNBC patients and non-basal/TNBC patients. **c** Molecular subtypes of RBM15 in the indicated cohorts. **d** Gene alteration of RBM15 in the TCGA-BRCA cohort. **e**–**g** IHC staining of breast cancer tissue specimens from a TMA slide was performed with an RBM15 antibody. Representative images of IHC staining of the specimens (**e**, **f**) and a graph of the results of IHC quantification (**g**). Student’s *t* test was applied for statistical significance (*p < 0.05, ***p < 0.005). **h**, **j** Patients in the indicated BC cohorts or basal/TNBC-specific cohorts were dichotomized by relatively high or relatively low RBM15 expression and were considered for plotting. The differences between these groups were significant as indicated (log‐rank test). (**i**, **k**) ROC curve analysis of the RBM15 expression-related probability of recurrence in the BC cohort (**i**) and basal/TNBC cohort (**k**). ROC curve analysis was performed to evaluate the correlation of RBM15 gene expression levels with overall survival by determining the area under the curve (AUC), which was estimated through the concordance index. The corresponding *p* values were determined using one-sided Wilcoxon signed-rank tests.

Previous research has suggested that m^6^A methyltransferases have oncogenic properties and contribute to tumorigenesis^5^. However, correlations between m^6^A methyltransferase expression and molecular subtypes in BC cells have not been identified. Hence, we explored which m^6^A methyltransferases are associated with different molecular subtypes of BC, including basal-like, Her2-enriched, luminal A, luminal B, and normal-like subtypes. As basal-like tumors are the dominant subset of TNBC tumors (ER; estrogen receptor, PR; progesterone receptor, and Her2 negative) that exhibit the worst prognosis, we performed a comparative analysis to determine which m^6^A methyltransferases are differentially expressed between the basal-like and non-basal-like subtypes, as described previously^27^. We found that the expression of RBM15 was increased in basal-like tumors in three independent patient cohorts (Fig. 1a). Using other BC cohorts, we confirmed that RBM15 expression was greater in basal-like and TNBC tumors than in non-basal-like tumors (Fig. 1b and Supplementary Fig. 1). In addition, RBM15 expression was significantly greater in basal-like BC/TNBC cell lines than in non-basal-like tumor BC/non-TNBC cell lines (Supplementary Fig. 2). As expected, among all the subtypes, RBM15 expression was the highest in the basal-like tumors (Fig. 1c and Supplementary Fig. 3). We also confirmed that RBM15 expression was significantly greater in breast tumor tissues than in normal tissues (Supplementary Fig. 4). As genes with oncogenic potential are genetically altered, we investigated RBM15 gene alterations in the TCGA-BRCA cohort. As shown in Fig. 1d, RBM15 was genetically altered in ~10% of BC tissue specimens; the most common alteration was gene amplification in basal-like tumors (Fig. 1d and Supplementary Fig. 5). Consistent with these findings, immunohistochemical (IHC) staining of human BC tissue specimens and normal human breast tissue specimens from the same patients revealed that RBM15 expression levels were much higher in BC tissue specimens than in normal breast tissue specimens (Fig. 1e–g) and that TNBC cells exhibited much higher RBM15 expression than non-TNBC cells (Fig. 1f, g).

As RBM15 expression was elevated in the BC molecular subtypes with the worst outcomes, we examined the association of RBM15 expression with clinical outcomes. Patients with higher RBM15 expression had worse survival outcomes than those with lower RBM15 expression (Fig. 1h and Supplementary Fig. 6). ROC curve analysis further supported the association of elevated RBM15 expression with worse prognosis in BC patients (Fig. 1i and Supplementary Fig. 7). As RBM15 expression was greater in basal-like BC than in non-basal-like BC and its association with outcomes could indicate increased RBM15 expression in basal-like TNBC, we also performed survival analysis of RBM15 expression in patients with basal-like BC/TNBC. Importantly, we found that RBM15 expression was associated with patient survival in basal-like BC/TNBC patients, indicating that the prognostic effects of RBM15 are not solely driven by tumor subtype (Fig. 1j, k, and Supplementary Fig. 8).

### RBM15 exhibits oncogenic properties in BC

**Fig. 2:**
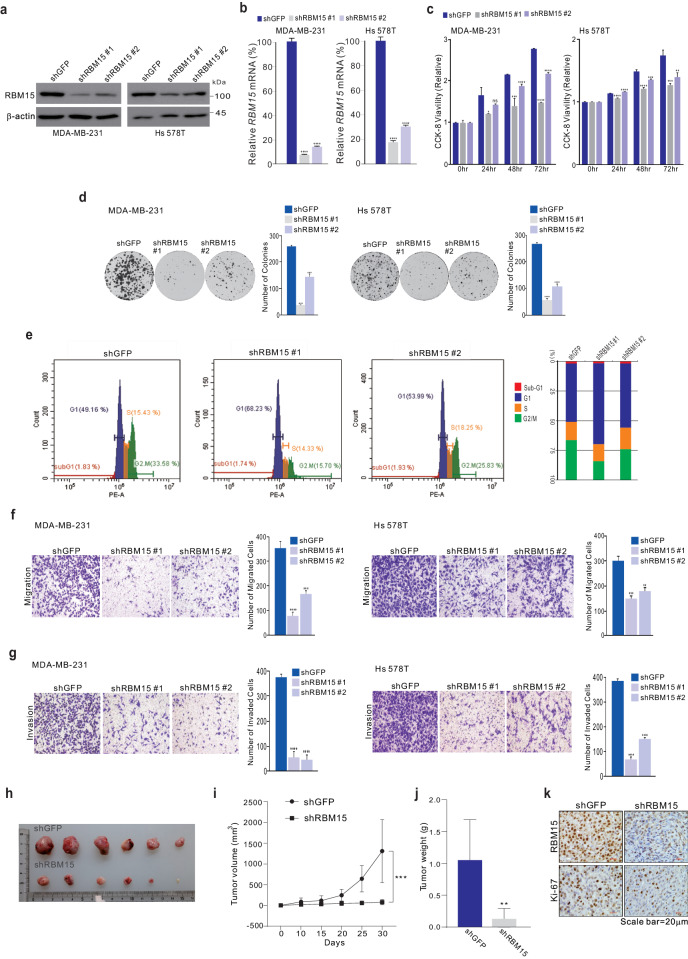
RBM15 contributes to the growth and motility of TNBC cells. **a**–**g** The indicated TNBC cells were stably transfected with shRBM15 or control shRNA (shGFP) and analyzed by western blotting (WB) analysis using the indicated antibodies (**a**) and by qRT‒PCR analysis (**b**). The infected cells were analyzed by a proliferation assay (CCK-8 assay) (**c**), a colony formation assay (**d**), and FACS analysis (**e**). The infected cells were subjected to cell migration assays using Boyden chambers (**f**). Boyden chamber Transwell assays were conducted without ECM for 36 h, and the migratory capacity of the cells was quantified by counting the number of stained cells. Cell invasion was analyzed using Boyden chambers, with Matrigel functioning as the ECM (**g**). The cells in the invasion assay were incubated for 36 h at 37 °C and stained with crystal violet. All the cells were quantified. **h**–**k** Xenograft experiments. MDA-MB-231 cells were infected with shRBM15 or control shRNA (shGFP) and selected with puromycin. After the infected MDA-MB-231 cells were injected into nude mice (**h**), the tumor volumes (**i**) and weights (**j**) were measured (*n* = 10). A representative IHC analysis of mouse samples was performed (**k**). All results are expressed as the means ± standard deviations (SDs) from three independent replicates (*p < 0.05, **p < 0.01, ***p < 0.005, and ****p < 0.001).

As RBM15 expression was correlated with clinical outcomes, we investigated the effects of RBM15 on the pathophysiology of BC model systems. Knockdown of RBM15 with short hairpin RNA (shRNA) in MDA-MB-231 and Hs 578 T TNBC cell lines resulted in reduced cell proliferation (Fig. 2a–c) and colony formation (Fig. 2d). In contrast, RBM15 overexpression increased colony formation (Supplementary Fig. 9). RBM15 knockdown increased the proportion of cells in the G1 phase and decreased the proportion of cells in the G2-M phase, consistent with its effects on cell proliferation (Fig. 2e). Similarly, this treatment significantly decreased cell migration and invasion (Fig. 2f, g). Importantly, RBM15 knockdown inhibited the growth of MDA-MB-231 xenografts, as evidenced by reduced tumor volume and weight (Fig. 2h–j). In addition, RBM15 knockdown downregulated the cell proliferation indicator Ki-67 (Fig. 2k). Thus, RBM15 plays important roles in TNBC proliferation, migration, and invasion in vitro and in tumor growth in vivo.

### RBM15 regulates genes involved in cancer metabolism in BC cells

**Fig. 3:**
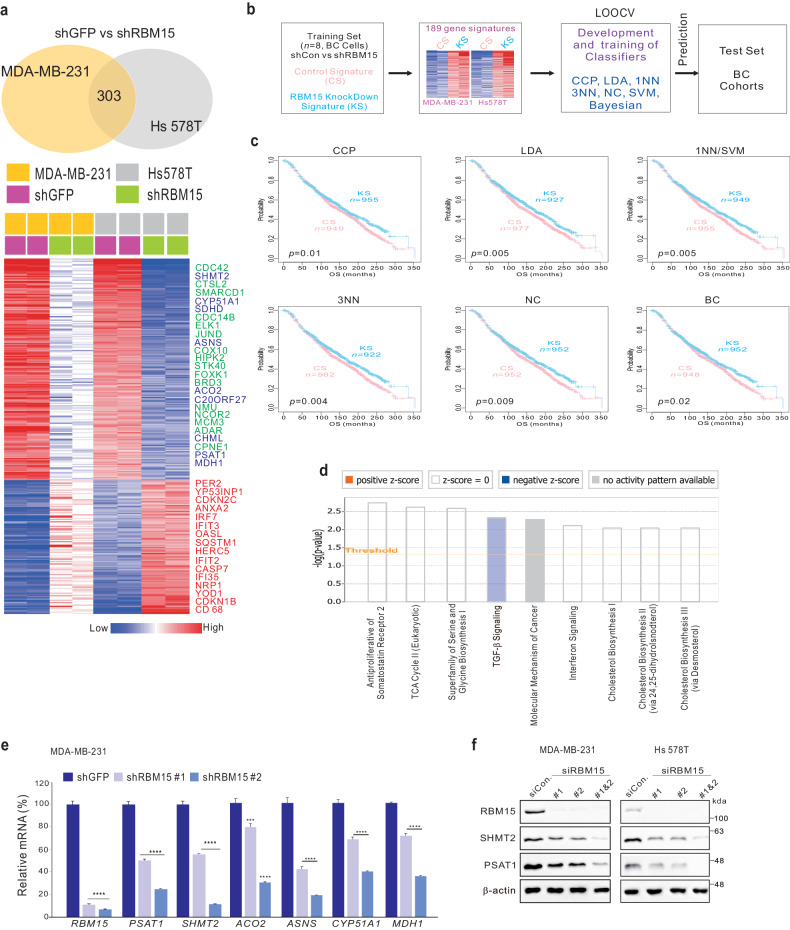
RBM15 gene signatures are associated with cancer metabolism. **a** Gene expression signatures specific to the loss of RBM15 expression via shRBM15 in two TNBC cell lines. Genes in the Venn diagram were selected by applying class comparison analysis via the BRB array tool (*p* < 0.001). The gene expression profiles are presented in matrix format. In this matrix, red and blue indicate relatively high and low expression levels, respectively, as indicated in the scale bar (log_2_-transformed scale). Genes associated with oncogenic potential are listed. **b** Schematic diagram of prediction model generation and evaluation of predicted outcomes based on a differentially expressed gene signature of RBM15 in BC cells. **c** A differentially expressed gene signature was used to construct a series of classifiers that estimated the probability of how much the expression pattern of BC patients was similar to the shared signature; control signature (CS) vs. knockdown signature (KS). K‒M plots of the OS of breast cancer patients in the TCGA-BRCA cohort were generated using the gene expression signature as a classifier. The differences between groups were significant as indicated (log-rank test). LOOCV leave-one-out cross-validation, CCP compound covariate predictor, 1NN one nearest neighbor, 3NN three nearest neighbors, NC nearest centroid, SVM support vector machine, LDA linear discriminator analysis. **d** Ingenuity pathway analysis (IPA) of genes differentially expressed after RBM15 silencing. **e**, **f** The indicated cells were infected or transfected with the indicated shRNA or siRNA. The cells were used for qRT‒PCR (**e**) and western blot (**f**) analyses of RBM15-associated genes in TNBC cells. The data are expressed as the means ± standard deviations (SDs) from three independent replicates. Student’s *t* test was performed to determine statistical significance (*p < 0.05, **p < 0.01, ***p < 0.005, and ****p < 0.001).

We next aimed to elucidate how RBM15 contributes to the pathogenesis of BC. Gene expression profiling of RBM15-silenced BC cells revealed that the expression levels of multiple genes were altered (Fig. 3a). Interestingly, genes with oncogenic potential, including *CDC42*, *CDC14B*, *STK40*, *BRD3*, *CPNE1*, and *MCM3*, were downregulated, whereas tumor inhibitors, including *CDKN2C*, *CASP7*, and *CDKN1B*, were upregulated by RBM15 depletion (Fig. 3a). As RBM15 predicts patient prognosis, we subsequently assessed whether the RBM15 gene signature is also correlated with clinical outcomes (Fig. 3b). Patients with tumors with a higher RBM15 signature (CS; control signature) had a worse prognosis than those with tumors with an RBM15 knockdown signature (KS) (Fig. 3c and Supplementary Fig. 10).

Next, to assess the effects of RBM15 gene regulation, we performed gene network analysis, as described previously^27^. Interestingly, the RBM15 gene network was associated with oncogenic pathways such as the TGF and interferon signaling pathways (Fig. 3d). In addition, cancer metabolism-related genes involved in serine and glycine metabolism, the TCA cycle, and cholesterol metabolism were included in the gene network. As shown in Fig. 3e, qRT‒PCR analysis revealed that depletion of RBM15 led to decreased expression of multiple genes related to metabolism (*PSAT1*, *SHMT2*, *ACO2*, *ASNS*, *CYP51A1*, and *MDH1*) (Fig. 3e). Furthermore, silencing RBM15 using small interfering RNA (siRNA) decreased the SHMT2 and PSAT1 protein levels (Fig. 3f).

### RBM15 regulates de novo serine and glycine synthesis to modulate BC cell proliferation

**Fig. 4:**
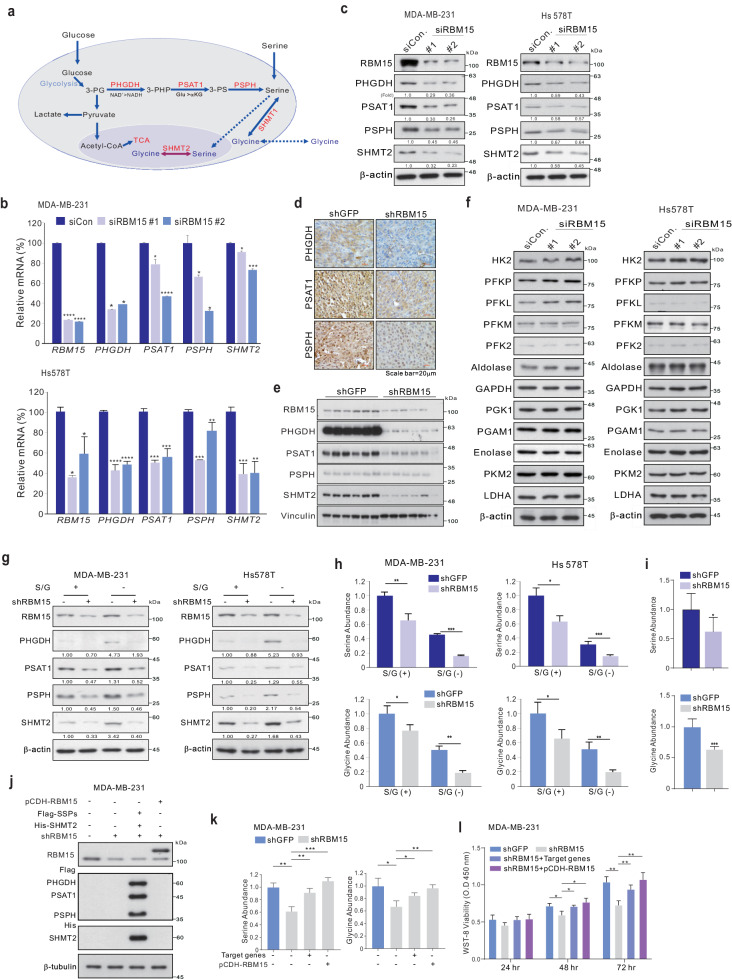
RBM15 regulates serine and glycine metabolism to induce BC cell growth. **a** A diagram depicting serine and glycine metabolism. **b**, **c**, **f** The indicated TNBC cells were transfected with siCon or siRBM15 and subjected to qRT‒PCR analysis with the indicated primers (**b**) and WB analysis (**c**, **f**) with the indicated antibodies. **d**, **e** IHC (**d**) and WB (**e**) analyses of RBM15 target genes in the mouse samples used in Fig. 2h. The indicated TNBC cells were infected with shRBM15 or shGFP. The cells were maintained for 5 days. The cells were used for WB analysis (**g**) and for detecting serine and glycine levels using colorimetric kits (**h**) (S/G: serine and glycine). **i** Serine and glycine metabolite levels were measured in the mouse samples used in Fig. 2h. **j**–**l** MDA-MB-231 cells were infected with shRBM15 or shGFP and transfected with Flag or His-tagged SSP cDNA. The cells were subjected to WB analysis with the indicated antibodies (**j**), serine and glycine assays (**k**), and WST-8 assays (**l**). The data are expressed as the means ± standard deviations (SDs) from three independent replicates. Student’s *t* test was performed to determine statistical significance (*p < 0.05, **p < 0.01, ***p < 0.005, and ****p < 0.001).

Gene expression profiling revealed that RBM15 regulates genes involved in serine and glycine metabolism. We extended these observations by analyzing genes related to the de novo serine synthesis pathway (SSP) (Fig. 4a). Interestingly, RBM15 silencing significantly decreased the mRNA (Fig. 4b) and protein (Fig. 4c) levels of PHGDH, PSAT1, PSPH, and SHMT2 in BC cells (Fig. 4b, c). In addition, the in vivo xenograft model revealed that RBM15 knockdown decreased the expression of SSP-related genes (Fig. 4d, e). However, the expression levels of glycolysis-related genes were not affected by RBM15 depletion (Fig. 4f), suggesting the selectivity of RBM15 for serine and glycine metabolism. In contrast, silencing of SSP-related genes, such as PHGDH, PSAT1, and PSPH, did not influence RBM15 expression (Supplementary Fig. 11), demonstrating that RBM15 functions as an upstream regulator of the serine and glycine metabolic pathway.

Serine and glycine deprivation increases the expression of SSP-related genes to activate SSP flux^9^. In BC cells, the protein expression level of SSP, but not that of RBM15, was elevated under serine and glycine deprivation (Fig. 4g). RBM15 depletion largely decreased the expression of SSP-related genes under both normal and serine- and glycine-deprived conditions in BC cells (Fig. 4g). Importantly, intracellular serine and glycine levels were significantly decreased by RBM15 depletion under both normal and serine- and glycine-deprived conditions, likely due to the inhibition of endogenous serine and glycine biosynthesis (Fig. 4h, i, Supplementary Fig. 12a, and Supplementary Fig. 12b). Ectopic expression of SSP-related genes (PHGDH, PSAT1, PSPH, and SHMT2, Fig. 4j) reversed the decreases in intracellular serine and glycine levels (Fig. 4k) and cell proliferation (Fig. 4l and Supplementary Fig. 12c–e) induced by RBM15 depletion. Taken together, these results indicate that RBM15 can promote serine and glycine biosynthesis and BC cell proliferation by upregulating SSP gene expression.

### RBM15 regulates serine and glycine metabolic genes by directly binding to their target RNA

**Fig. 5:**
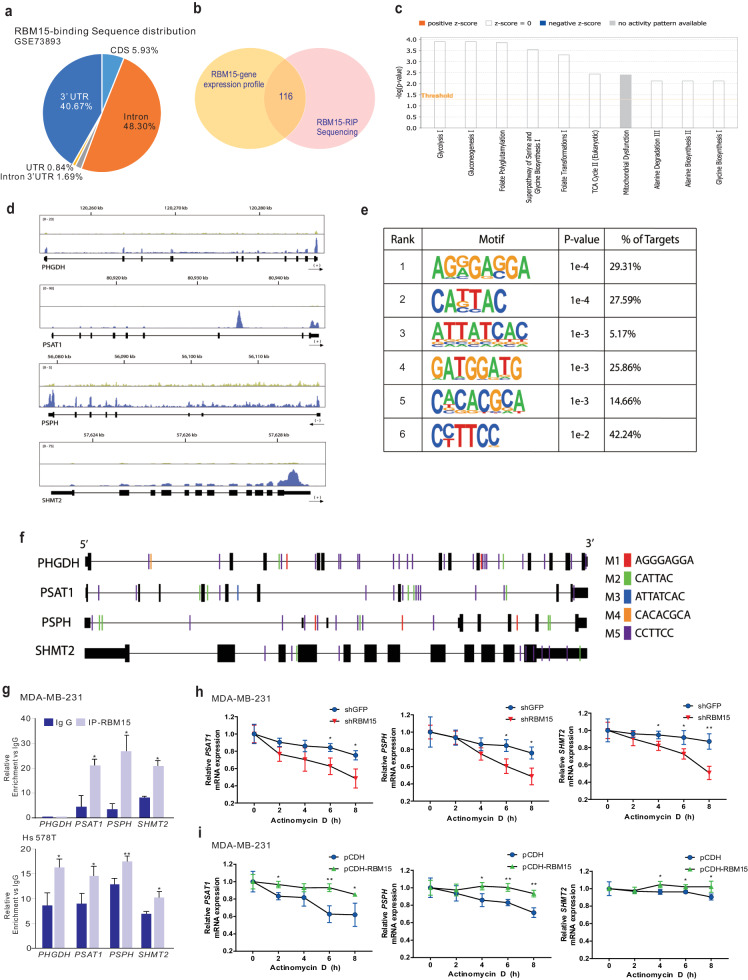
RBM15 directly regulates genes involved in serine and glycine metabolism in BC patients. **a** RIP-seq data (GSE73893) were processed for sequence distribution. **b** Comparison of gene expression data and RIP-seq data using a Venn diagram. **c** IPA analysis with comparison data. **d** UCSC genome browser view of RIP-seq reads mapped to PHGDH, PSAT1, PSPH, and SHMT2. **e** Significantly enriched RNA motifs among the RIP-seq data. **f** Sequence alignment of the PHGDH, PSAT1, PSPH, and SHMT2 genomic loci based on the RBM15 binding motif. **g** RIP analyses of MDA-MB-231 and Hs 578 T cells were performed using the indicated antibodies. Next, qRT‒PCR analysis was performed using primers against the PHGDH, PSAT1, PSPH, and SHMT2 mRNAs. **h**, **i** MDA-MB-231 cells were infected with shRBM15 or shGFP (**h**) and pCDH-RBM15 or empty vector (**i**). After infection, the cells were treated with DMSO or actinomycin D (Act D) and harvested at the indicated time points. Total RNA was extracted from the indicated cells and analyzed by qRT‒PCR with the indicated primers. The RNA level is designated the RNA half-life. All results are expressed as the means ± standard deviations (SDs) from replicates (*p < 0.05, **p < 0.01, ***p < 0.005, and ****p < 0.001).

m^6^A methyltransferases have been proposed to bind to downstream target RNAs^4,8^. m^6^A methylation can alter RNA splicing, translation, and stability^5^. Previous genomic analysis by RNA immunoprecipitation sequencing (RIP-seq) revealed numerous RBM15 target RNAs^20,28^. Our reanalysis of RIP-seq data revealed that RBM15 primarily bound to introns and UTRs (Fig. 5a), as reported previously for other RBPs and m^6^A methyltransferases^29^. We integrated RBM15 gene expression data (Fig. 3) and RIP-seq data to identify genes that are directly regulated by RBM15 (Fig. 5b). Network analysis of potential target RNAs of RBM15 demonstrated that metabolism-related pathways (glycolysis, gluconeogenesis, folate metabolism, serine and glycine biosynthesis, and the TCA cycle) were highly enriched (Fig. 5c). Based on the potential role of RBM15 in regulating serine and glycine synthesis through the expression of SSP-related genes, we explored potential RBM15 binding sites on PHGDH, PSAT1, PSPH, and SHMT2. Binding peak and motif analyses revealed RBM15 binding regions and specific binding sites on PHGDH, PSAT1, PSPH, and SHMT2 (Fig. 5d–f and Supplementary Fig. 13). Next, to validate whether RBM15 directly bound to target genes, we performed RIP experiments in BC cells and confirmed the binding of RBM15 to PHGDH, PSAT1, PSPH, and SHMT2 RNAs in these cells (Fig. 5g). To determine whether RBM15 affects the stability of bound RNA, we measured the RNA levels in actinomycin D-treated cells with RBM15 silencing or overexpression and found that the stability of SSP mRNAs (*PSAT1*, *PSPH*, and *SHMT2*) was significantly decreased by RBM15 silencing (Fig. 5h and Supplementary Fig. 14b) or increased by RBM15 overexpression (Fig. 5i and Supplementary Fig. 14d) without affecting *PHGDH* mRNA stability (Supplementary Fig. 14). Our results indicated that RBM15 binds to SSP mRNAs and modulates their stability, which potentially contributes to the oncogenic activity of RBM15.

### RBM15 mediates m^6^A modification on target mRNAs of SSP genes

The role of RBM15 in m^6^A modification is largely unknown. We assessed the correlation between m^6^A activity and RBM15 levels in BC cells.

**Fig. 6:**
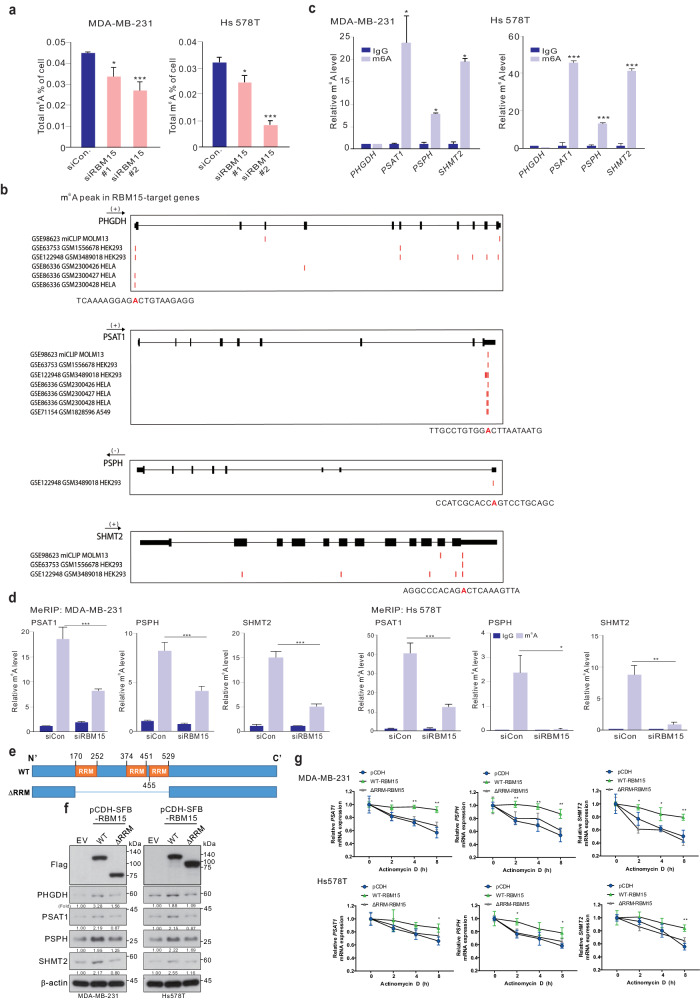
RBM15 regulates the m^6^A modification of serine and glycine metabolic genes. **a** The m^6^A content of total RNA in MDA-MB-231 and Hs578T BC cells with or without RBM15 silencing was determined. **b** The MeRIP-seq peaks of the indicated genes from the Gene Expression Omnibus were aligned. The methylated adenosine base is denoted with a red box and red “A”. **c** Methylated RNA in MDA-MB-231 and Hs578T cells. **d** MDA-MB-231 and Hs 578 T cells were transfected with siRBM15 or siCon and immunoprecipitated with a m^6^A antibody. Next, qRT‒PCR analysis was performed using primers against the PSAT1, PSPH, and SHMT2 mRNAs. **e**–**g** Diagram of the RBM15 mutant construct. **e** MDA-MB-231 and Hs578T cells were stably infected with SFB (S protein, Flag, and streptavidin-binding peptide)-tagged WT RBM15 or ΔRRM-RBM15. The infected cells were harvested, and protein expression levels were analyzed by WB using the indicated antibodies (**f**). Then, the infected cells were treated with DMSO or actinomycin D (Act D) and harvested at the indicated time points for qRT‒PCR with the indicated primers (**g**). The data are expressed as the means ± SDs of triplicate samples (*p < 0.05, **p < 0.01, ***p < 0.005, and ****p < 0.001).

RBM15 silencing significantly reduced the total m^6^A abundance in MDA-MB-231 and Hs 578 T cells (Fig. 6a). Sequence alignment revealed that adenosine bases within the exons and UTRs of the PHGDH, PSAT1, PSPH, and SHMT2 mRNAs were methylated (Fig. 6b), indicating that downstream target mRNAs could undergo RBM15-mediated methylation. Further immunoprecipitation using an m^6^A-specific antibody and subsequent qRT‒PCR analysis revealed that RBM15-targeted SSP mRNAs were methylated in BC cells (Fig. 6c). Importantly, RBM15 silencing reduced the m^6^A levels in the mRNAs of PSAT1, PSPH, and SHMT2 (Fig. 6d). Our results indicate that RBM15 modulates the m^6^A levels of a suite of genes involved in serine and glycine metabolism in BC cells. As RBM15 functions as an m^6^A regulator, we investigated whether the RNA recognition motif (RRM) plays an important role in the RBM15-induced stability of SSP mRNAs using an RNA recognition motif (RRM)-deleted RBM15 mutant (ΔRRM-RBM15; Fig. 6e). While ectopic expression of wild-type (WT) RBM15 increased the stability of SSP mRNAs and the protein expression level of SSP, ΔRRM-RBM15 did not show these effects (Fig. 6f, g) in BC cells, suggesting that RBM15 specifically recognizes SSP mRNAs and influences their m^6^A levels and stability.

### RBM15 regulates SSP genes in a METTL3/14-dependent manner

**Fig. 7:**
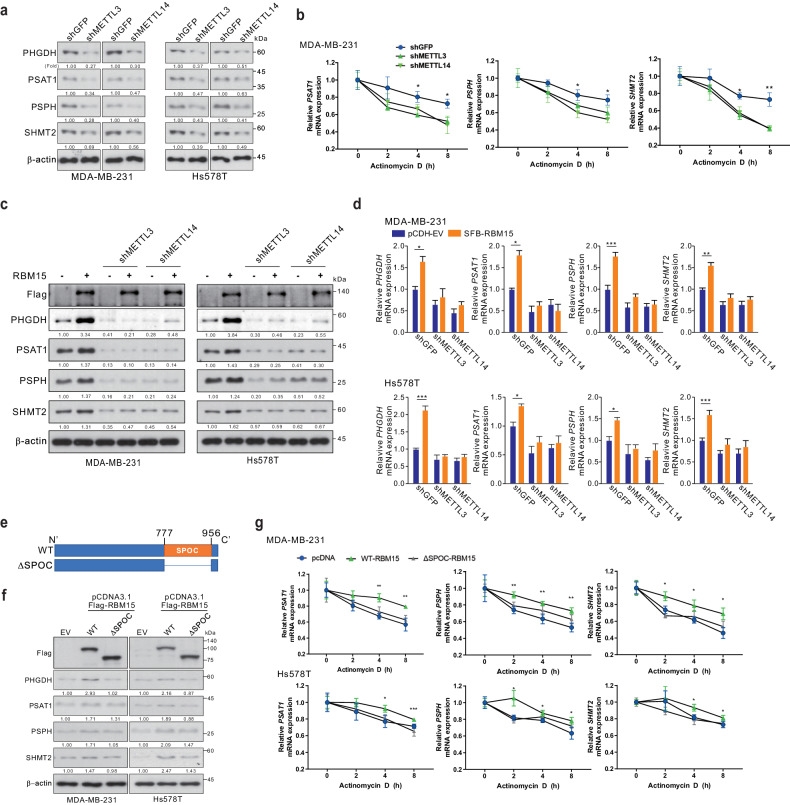
SSP gene regulation by RBM15 is dependent on the m^6^A writer METTL3/14. **a** MDA-MB-231 and Hs 578T cells were transfected with the indicated shRNAs, and the cell lysates were subjected to WB analysis with the indicated antibodies. **b** The infected cells were treated with DMSO or actinomycin D (Act D) and harvested at the indicated time points for qRT‒PCR. **c**, **d** MDA-MB-231 and Hs 578T cells were transfected with the indicated shRNAs and Flag-tagged RBM15 or empty vector. The infected cells were analyzed by WB (**c**) or qRT‒PCR (**d**) using the indicated antibodies and primers, respectively. **e**–**g** Diagram of the RBM15 mutant construct (**e**). MDA-MB-231 and Hs 578T cells were transfected with Flag-tagged RBM15 or empty vector and analyzed by WB using the indicated antibodies (**f**). The infected cells were treated with DMSO or actinomycin D (Act D) and harvested at the indicated time points for qRT‒PCR with the indicated primers (**g**). The data are expressed as the means ± SDs of triplicate samples (*p < 0.05, **p < 0.01, and ***p < 0.005).

RBM15 is a component of the m^6^A writer complex without methyltransferase activity. Thus, RBM15 may specifically recognize SSP RNAs and then guide them to METTL3/14, which have RNA methyltransferase activity, to induce m^6^A modification and SSP gene expression. To determine whether RBM15-induced SSP mRNA stability and expression are dependent on METTL3/14, we stably depleted METTL3/14 using shRNAs (Supplementary Fig. 15a). Consistent with the results with RBM15 depletion, the stability of SSP mRNAs and protein expression levels of SSP were significantly diminished by METTL3/14 depletion (Fig. 7a, b and Supplementary Fig. 15d), while silencing of the m^6^A readers IGF2BP1, 2, or 3 did not alter the overall SSP gene expression level, although some SSP genes showed slight changes (Supplementary Fig. 15a, c). Furthermore, we found that RBM15 overexpression-induced increases in the mRNA and protein expression levels of SSP were not detected in the absence of METTL3/14 (Fig. 7c, d and Supplementary Fig. 15b). These results indicate that RBM15 induces the expression of SSP genes in a METTL3/14-dependent manner. Previous reports demonstrated that the Spen ortholog and paralog C-terminal (SPOC) domain of RBM15 is important for maintaining the biological activity of RBM15 through the formation of a complex with METTL3/14^13^. Next, we investigated whether the SPOC domain of RBM15 is essential for SSP gene expression using a ΔSPOC-RBM15 mutant (Fig. 7e). As shown in Fig. 7f, g, while RBM15 WT overexpression increased the stability of SSP mRNAs and protein expression levels of SSP, the ΔSPOC-RBM15 mutant did not, suggesting that the SPOC domain in RBM15 is required for RBM15 function.

### Clinical association between RBM15 and SSP-related genes in BC

**Fig. 8:**
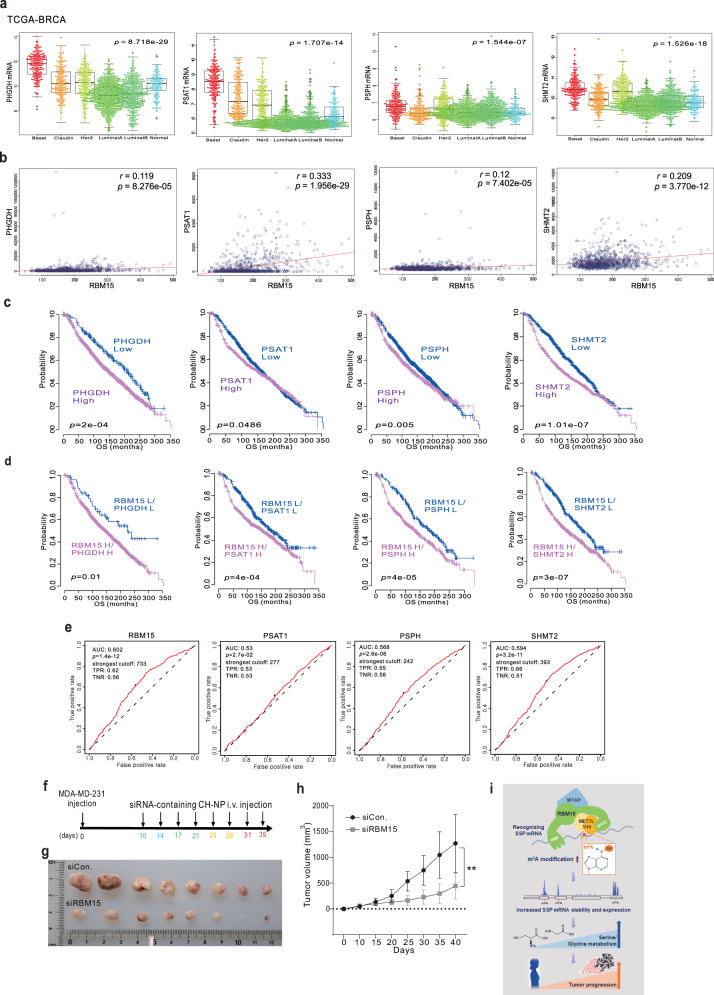
Clinical implications of RBM15 target genes and clinical efficacy of RBM15 in BC patients. **a** Molecular subtypes of PHGDH, PSAT1, PSPH, and SHMT2 in the TCGA-BRCA cohort. **b** Correlations between RBM15 and PHGDH, PSAT1, PSPH, and SHMT2 gene expression in the indicated TCGA-BRCA cohort. Scatter plots of RBM15 and related genes in the cohorts are shown for the indicated cohorts. **c**, **d** Kaplan‒Meier plots of OS outcomes in patients from the TCGA-BRCA cohort. **e** ROC curve analysis was performed to assess the correlation of RBM15 gene expression levels with chemotherapy response by determining the area under the curve (AUC), which was estimated through the concordance index. The corresponding *p* values were determined using one-sided Wilcoxon’s signed-rank test. **f**–**h** After MDA-MB-231 cells were injected into nude mice, the indicated siRNAs containing CH-NPs were administered according to the indicated treatment schedule (**f**). A representative image of a tumor is shown. **g** The tumor volumes were measured (**h**). **i** Schematic diagram of the RBM15 gene regulatory mechanism. All results are expressed as the means ± standard deviations of three independent replicates (*p < 0.05 and **p < 0.01).

To investigate the clinical relevance of the RBM15 target genes, including PHGDH, PSAT1, PSPH, and SHMT2, we performed genomic data analysis using the TCGA and GEO datasets. Similar to RBM15 expression, the expression levels of PHGDH, PSAT1, PSPH, and SHMT2 were elevated in basal-like BC subtypes (Fig. 8a). As expected, RBM15 expression was significantly correlated with PHGDH, PSAT1, PSPH, and SHMT2 expression in BC (Fig. 8b). These correlations were maintained in multiple BC cohorts, including TNBC cohorts (Supplementary Figs. 16 and 17). We also noted genetic alterations in PHGDH, PSAT1, PSPH, and SHMT2 in the TCGA cohort (Supplementary Fig. 18). As shown in Fig. 8c, similar to RBM15 expression, elevated expression levels of PHGDH, PSAT1, PSPH, and SHMT2 were correlated with a worsened outcome in BC patients. Furthermore, high expression levels of RBM15, PHGDH, PSAT1, PSPH, and SHMT2 were correlated with a worse prognosis in BC patients (Fig. 8d, Supplementary Fig. 19a, 19b) and TNBC patients (Supplementary Fig. 19c,d). Chemotherapy responsiveness is a major factor determining outcomes in BC patients. ROC curve analysis demonstrated that RBM15, PSAT1, SHMT2, and PSPH were significantly associated with chemotherapy response (Fig. 8e). These results suggest that RBM15 and RBM15 target genes are associated with worsened clinical outcomes by conferring drug resistance. Overall, our data indicate that RBM15 contributes to tumorigenesis by increasing serine and glycine metabolism and that RBM15 could be a promising therapeutic target for TNBC patients. Hence, to determine the therapeutic efficacy of RBM15 targeting, we targeted RBM15 using a siRNA-mediated delivery method, which is widely used for gene knockdown in cancer cells. As expected, siRBM15 significantly reduced tumor growth and weight (Fig. 8f–h). Furthermore, the levels of RBM5 target genes were decreased (Supplementary Fig. 20).

## Discussion

m^6^A is the most common RNA modification and contributes to molecular and cellular functions in mammals^2^. Recently, multiple studies have suggested that m^6^A modification confers oncogenic properties and potentially influences patient outcomes, including survival and drug responsiveness^5^. However, the precise role of RBM15 as an m^6^A regulator in these processes, including cancer, has not been studied in detail.

In this study, we examined the role of RBM15 in BC cells and found that it specifically targets SSP genes to induce m^6^A modification and the expression of SSP and subsequent de novo serine and glycine biosynthesis and tumor progression (Fig. 8i). RBM15 has been implicated in the transcriptional repression of the XIST gene, leading to X-chromosome inactivation^30^. This molecule is also involved in HCC progression via the IGF2BP1-YES1-MAPK axis^31^. While genomic screening using CRISPR-Cas9 revealed that disrupting the m^6^A methyltransferase YTHDF2 can trigger apoptosis in TNBC models^6^, the clinical implications and molecular mechanisms underlying the effects of YTHDF2 have not been fully elucidated. In the present study, we found that RBM15 expression was elevated in the basal-like BC/TNBC subtype and was associated with clinical outcomes (Fig. 1). Previous studies revealed that another RNA-binding protein (RBP), albeit not an m^6^A methyltransferase, is highly expressed in TNBC cells and influences cell growth through STAT3 in BC cells, similar to RBM15^27^. Thus, RNA-related molecules can play a crucial role in TNBC. As RBM15 expression is TNBC-specific, RBM15 targeting may be a novel therapeutic option for TNBC.

Network analysis revealed that RBM15 depletion was implicated in serine and glycine metabolism (Fig. 3). Recently, elevated serine and glycine biosynthesis has been proposed to promote tumorigenesis^9^. In general, rapidly growing cells selectively take up exogenous serine or synthesize serine via the SSP to alter intracellular glycine and one-carbon units for nucleotide biosynthesis. PHGDH, PSAT1, PSPH, and SHMT2 are major players contributing to the SSP, which is a crucial metabolic network that causes tumorigenesis and is clinically important^32^. Thus, SSP targeting is emerging as a novel therapeutic approach for cancer. Our investigation confirmed that RBM15 is an important upstream factor regulating SSP-related genes. Thus, blocking the SSP by modulating RBM15 is an effective method to target all genes involved in the SSP.

We found that RBM15 bound to and enhanced the m^6^A RNA methylation of SHMT2, PSAT1, and PSPH but not that of PHGDH; these are key mediators of serine and glycine synthesis (Figs. 5 and 6). Interestingly, the m^6^A levels of PHGDH were very low, and RBM15 did not affect the stability of PHGDH. However, RBM15 influenced the gene expression level of PHGDH, suggesting that RBM15 regulates PHGDH expression in an m^6^A-independent manner. Further research is warranted to determine how RBM15 modulates target gene expression levels without affecting m^6^A modification.

The m^6^A reader YTHDF3^33^ and m^6^A eraser FTO^34^ have been found to regulate glycolysis. However, we found that RBM15 did not alter the expression of genes involved in glycolysis (Fig. 4f), suggesting that RBM15 has selective effects on serine and glycine metabolism. Potential agents that directly target serine and glycine synthesis have not been developed thus far. Therefore, targeting RBM15, which contributes to serine and glycine metabolism as an upstream regulator, could be a good alternative therapeutic approach to control serine and glycine metabolism for cancer treatment.

Although we focused on RBM15 and serine metabolism in this study, network analysis revealed that the relationship between RBM15 and other genes with oncogenic potential (CDC42, CDC14B, STK40, BRD3, CPNE1, and MCM3) is also crucial for cancer cell survival. The oncogenic axis involving RBM15 and potential oncogenic target genes should be identified in further research.

m^6^A is relatively uncommon and is found on 0.1–0.4% of adenosine residues in cellular RNA^1,4,35,36^. However, m^6^A increases with tumor stage and malignancy, suggesting that m^6^A modification plays an important role in tumorigenesis. Recently, a highly potent chemical inhibitor, STM2457, which disrupts the METTL3 and METTL14 complexes, was developed. This inhibitor is effective against myeloid leukemia^8^. STM2457 reduces m^6^A RNA methylation levels and causes mRNA translation defects, indicating that STM2457 is a bona fide METTL3 inhibitor. Thus, the development of selective RBM15 inhibitors that are effective against cancer is possible.

RBM15 is classified as an m^6^A writer similar to MELLT3/14, which have methyltransferase activity, in the m^6^A writer complex. Although whether RBM15 possesses methyltransferase activity is controversial, RBM15 is known to play a crucial role in regulating the mRNA stability and expression of SSP genes. Our data demonstrated that RBM15 directly bound to the target RNA (Fig. 5) and that the methylation of the target RNA was affected by RBM15 (Fig. 6), although the methylation of some SSP genes, such as PHGDH, was not mediated by RBM15. Based on previous reports, RBM15 is present in the complex of METTLs that regulate SSP genes and promotes METTL activity to regulate SSP genes. RBM15 silencing did not affect the m^6^A activity of PHGDG. Thus, RBM15 might regulate PHGDH expression in a methyltransferase activity-independent manner via the m^6^A writer complex.

As the genes involved in cancer metabolism contribute to drug resistance, the reprogramming of cancer metabolism has emerged as a potential therapeutic target^37^. As shown in Fig. 8, we found that PHGDH, PSAT1, PSPH, SHMT2, and RBM15 are associated with patient prognosis and chemotherapy resistance. RBM15 functions as an upstream regulator of the serine and glycine pathway, which is involved in chemotherapy resistance, suggesting that RBM15 targeting may be an effective approach to overcome chemotherapy resistance.

In conclusion, we showed that the m^6^A regulator RBM15 is an upstream regulator of serine and glycine metabolism. We propose RBM15 as a novel and attractive therapeutic target for drug development against BC. This molecule could act by impacting the metabolic changes required for cancer development and progression.

### Supplementary information

Supplementary information
