# Supplementary material for: The m6A writer RBM15 drives the growth of triple-negative breast cancer cells through the stimulation of serine and glycine metabolism
Source: Exp Mol Med. 2024 Jun 3;56(6):1373–87. doi: 10.1038/s12276-024-01235-w (PMC11263342; doi:10.1038/s12276-024-01235-w)
Supplement: Supplementary file 1 — Supplementary information [file 12276_2024_1235_MOESM1_ESM.pdf]

Supplementary Fig. 1

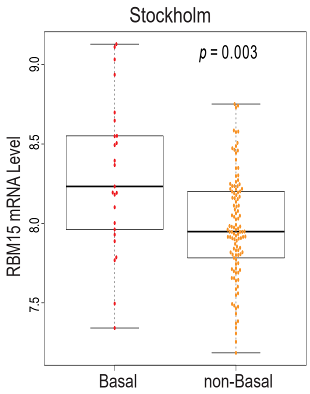

Supplementary Fig. 1| RBM15 expression in human breast cancer.

RBM15 expression level from indicated BC patient cohorts. RBM15 expression between basal and non-basal from indicated BC patients. *p*-value indicates significance of analysis.

Supplementary Fig. 2

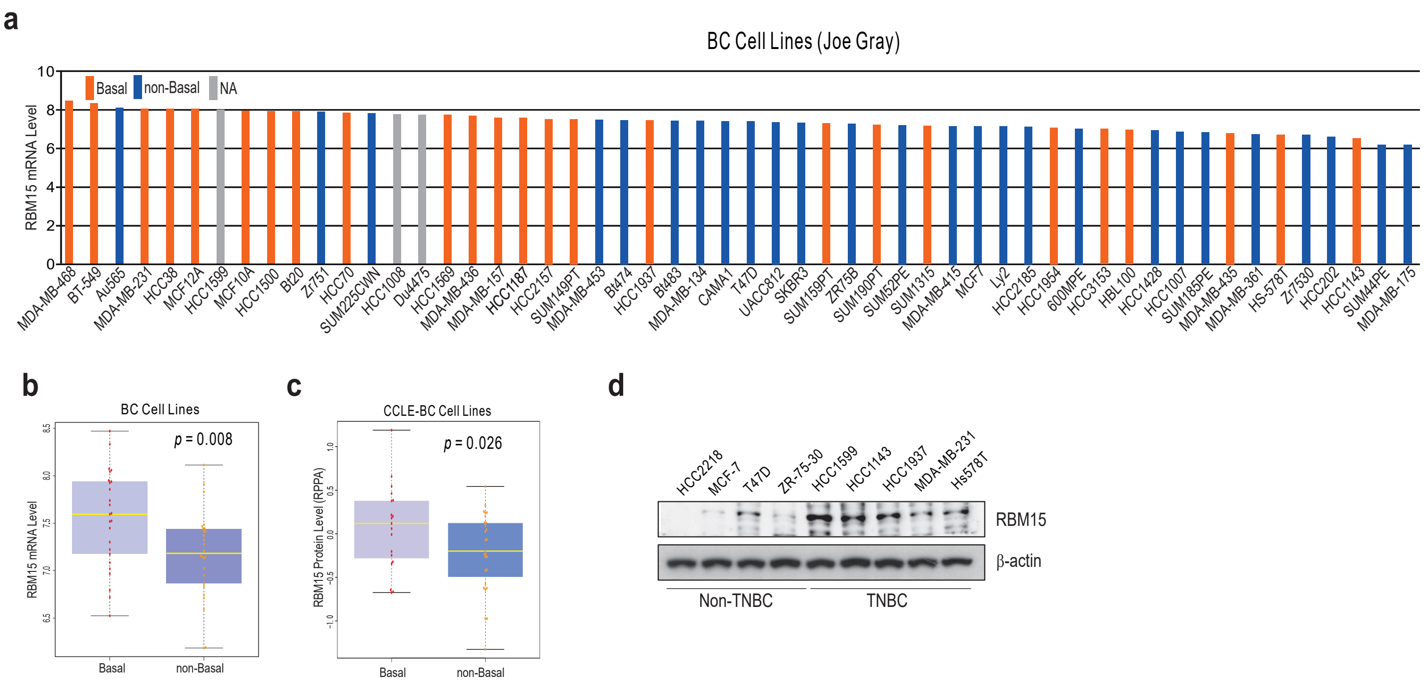

Supplementary Fig. 2| RBM15 expression in human breast cancer cell lines.

(a) RBM15 expression level from indicated BC cell lines. (b and c) RBM15 expression between basal and non-basal from indicated BC cell lines gene expression data sets. (d) Western blot analysis with indicated cells and antibodies. *p*-value indicates significance of analysis.

Supplementary Fig. 3

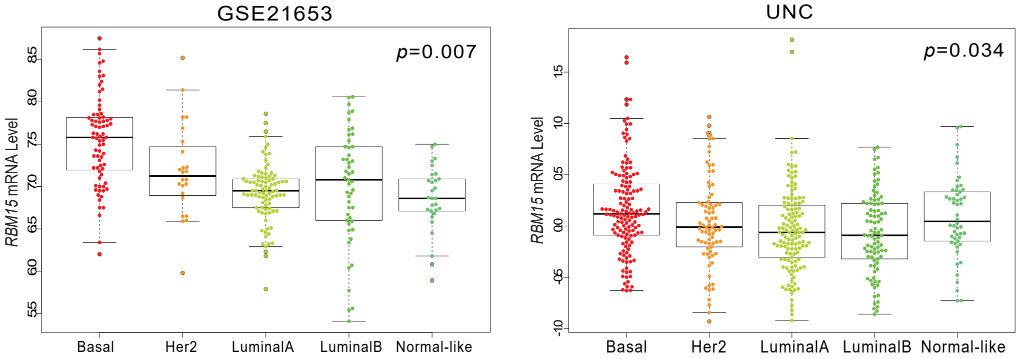

Supplementary Fig. 3| RBM15 expression in human breast cancer patients.

RBM15 expression from indicated BC cohorts-depending on molecular subtypes. *p*-value indicates significance of analysis.

Supplementary Fig. 4

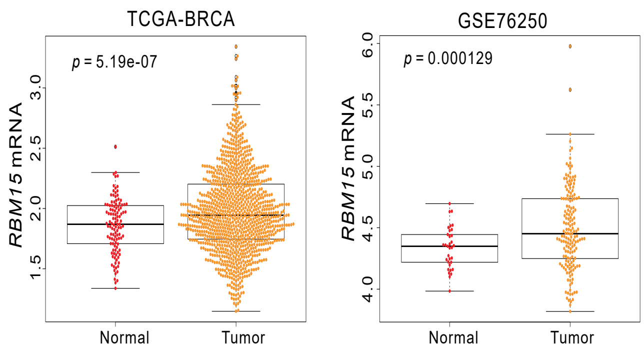

Supplementary Fig. 4| RBM15 expression in human breast cancer.

RBM15 expression between normal and tumor from indicated BC patients. *p*-value indicates significance of analysis.

Supplementary Fig. 5

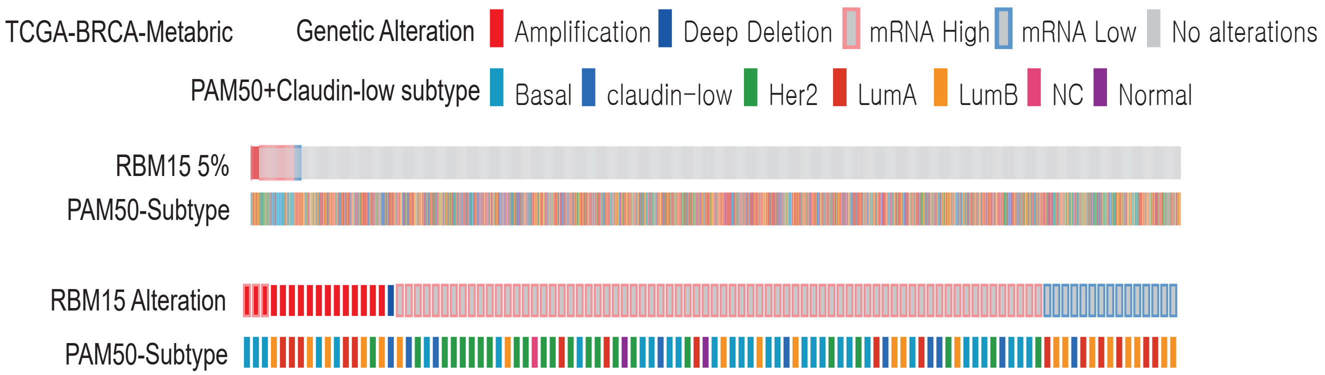

Supplementary Fig. 5| Genetic alteration of RBM15 in breast cancer.

Genetic alteration of RBM15 from TCGA-BRCA cohorts. These analysis were done in cBioportal.

Supplementary Fig. 6

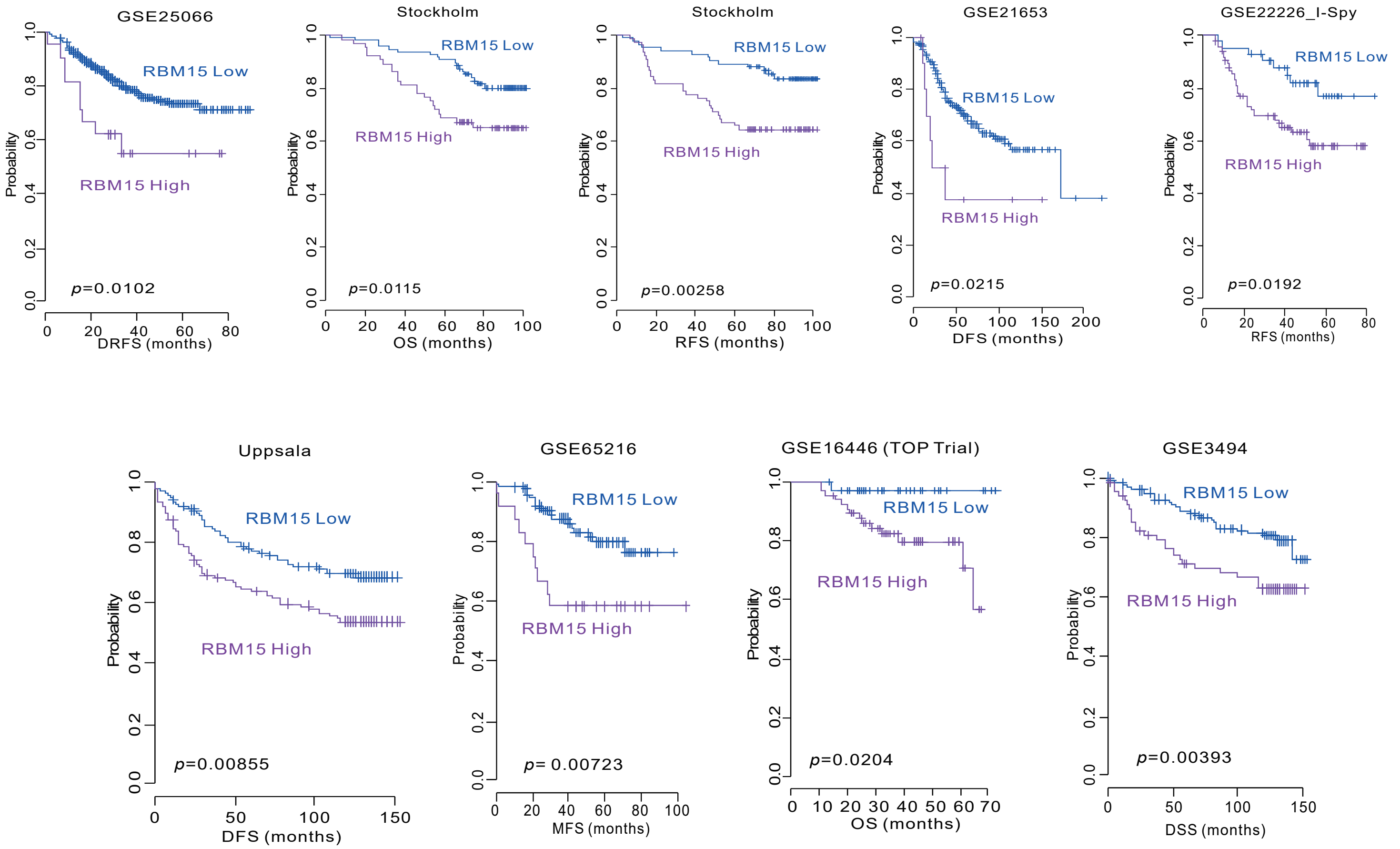

Supplementary Fig. 6| Survival analysis of RBM15 in human breast cancer

Using indicated cohort, patients were classified according to the expression level of RBM15. Kaplan-Meier plots and the log-rank test were used to estimate patient prognosis. *p*-value indicates significance of analysis.

Supplementary Fig. 7

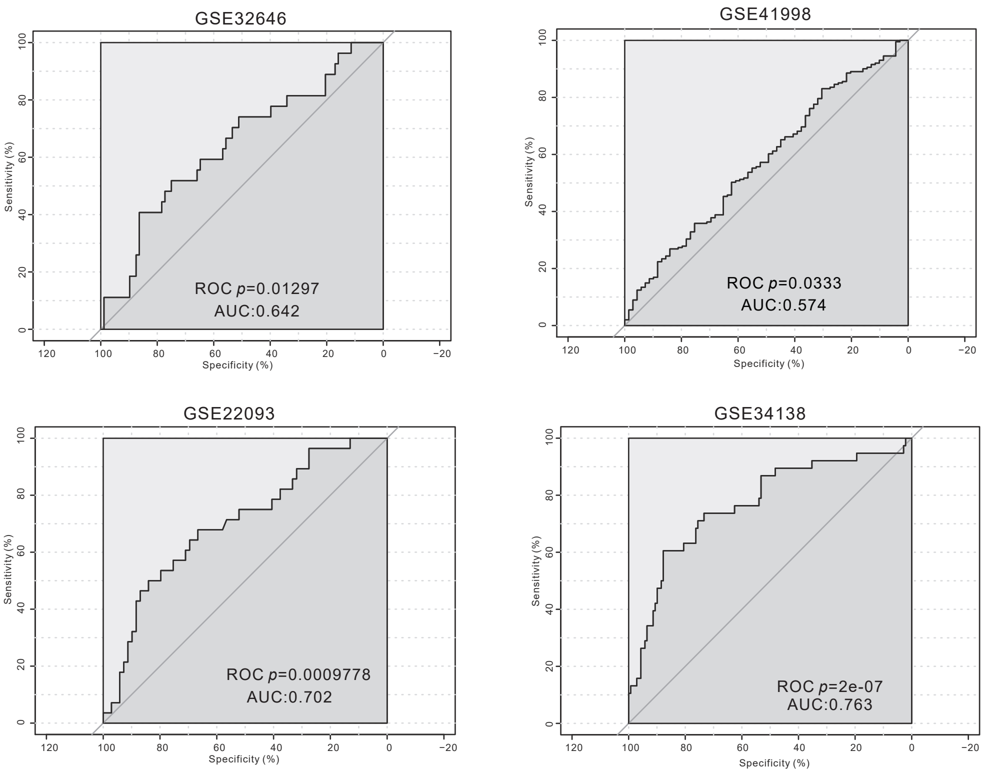

Supplementary Fig. 7| Survival analysis of RBM15 in human breast cancer

ROC (Receiver operating characteristic) analysis with indicated cohorts. *p*-value indicates significance of analysis.

Supplementary Fig. 8

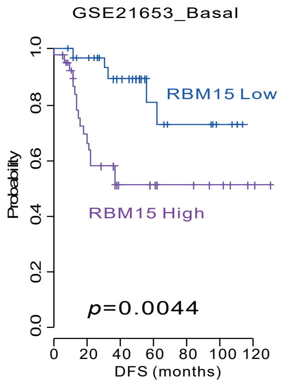

Supplementary Fig. 8| Survival analysis of RBM15 in basal BC

Using indicated cohort, patients were classified according to the expression level of RBM15. Kaplan-Meier plots and the log-rank test were used to estimate patient prognosis. *p*-value indicates significance of analysis.

Supplementary Fig. 9

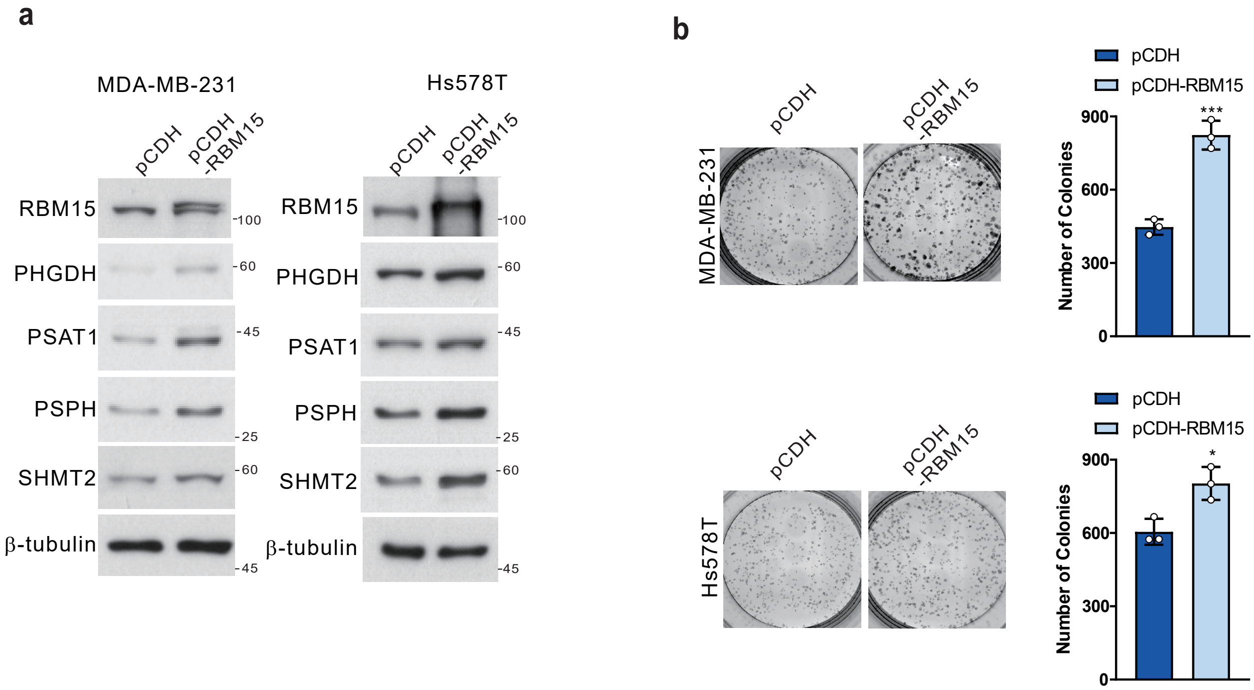

Supplementary Fig. 9| Target gene expression of RBM15 in BC

(a and b) MDA-MB-231 and Hs578T breast cells were infected with indicated cDNA pCDH or pCDH-RBM15). The cells were used for WB assay with indicated antibodies (a) and used for colony formation assay (b) (* $p<0.05$, ** $p<0.01$ and *** $p<0.005$).

Supplementary Fig. 10

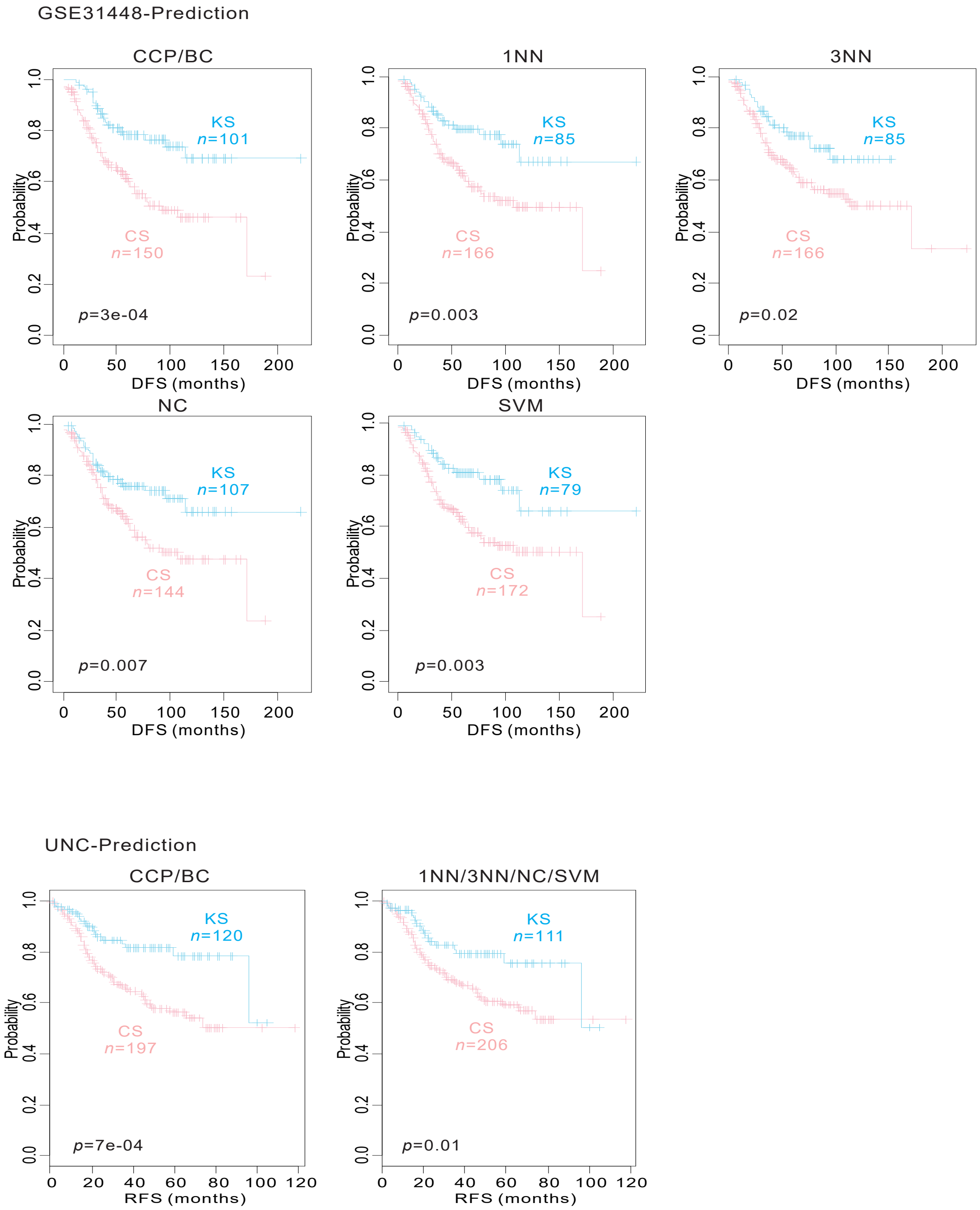

Supplementary Fig. 10| Survival prediction with RBM15 gene signatures in BC

Kaplan-Meier plots of DFS and RFS (Relapse-free survival) of BC patients from GSE31448 and UNC cohorts predicted by using RBM15 gene expression signature with indicated classifiers (CS:Cotrol Signature; KS: Knock down Signature; SC:Compound covariate predictor (CCP), one nearest neighbor (1NN), three nearest neighbor (3NN), nearest centroid (NC), support vector machines (SVM) and bayesian analysis (BC). *p*-value indicates significance between groups-based on log-rank test.

Supplementary Fig. 11

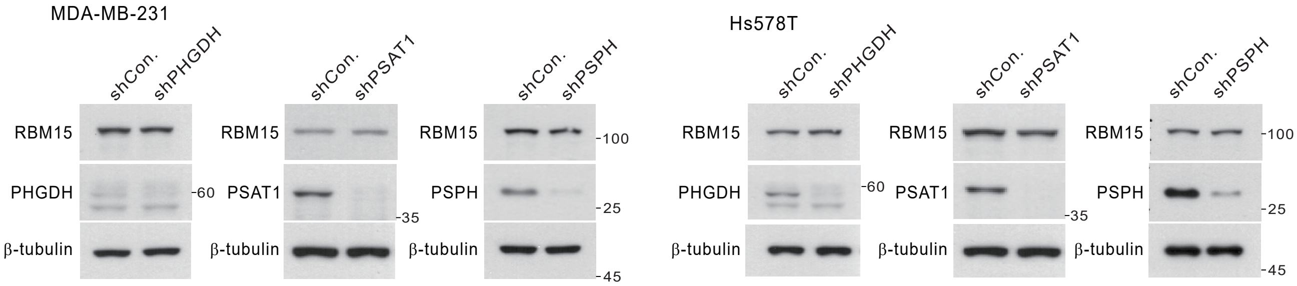

Supplementary Fig. 11| SSP-silencing effect on RBM15 gene expression BC

MDA-MB-231 (left) and Hs578T(right) breast cells were infected with indicated shRNAs and the cells were used for WB assay with indicated antibodies and used for colony formation assay (* p<0.05, ** p<0.01 and *** p<0.005).

Supplementary Fig. 12

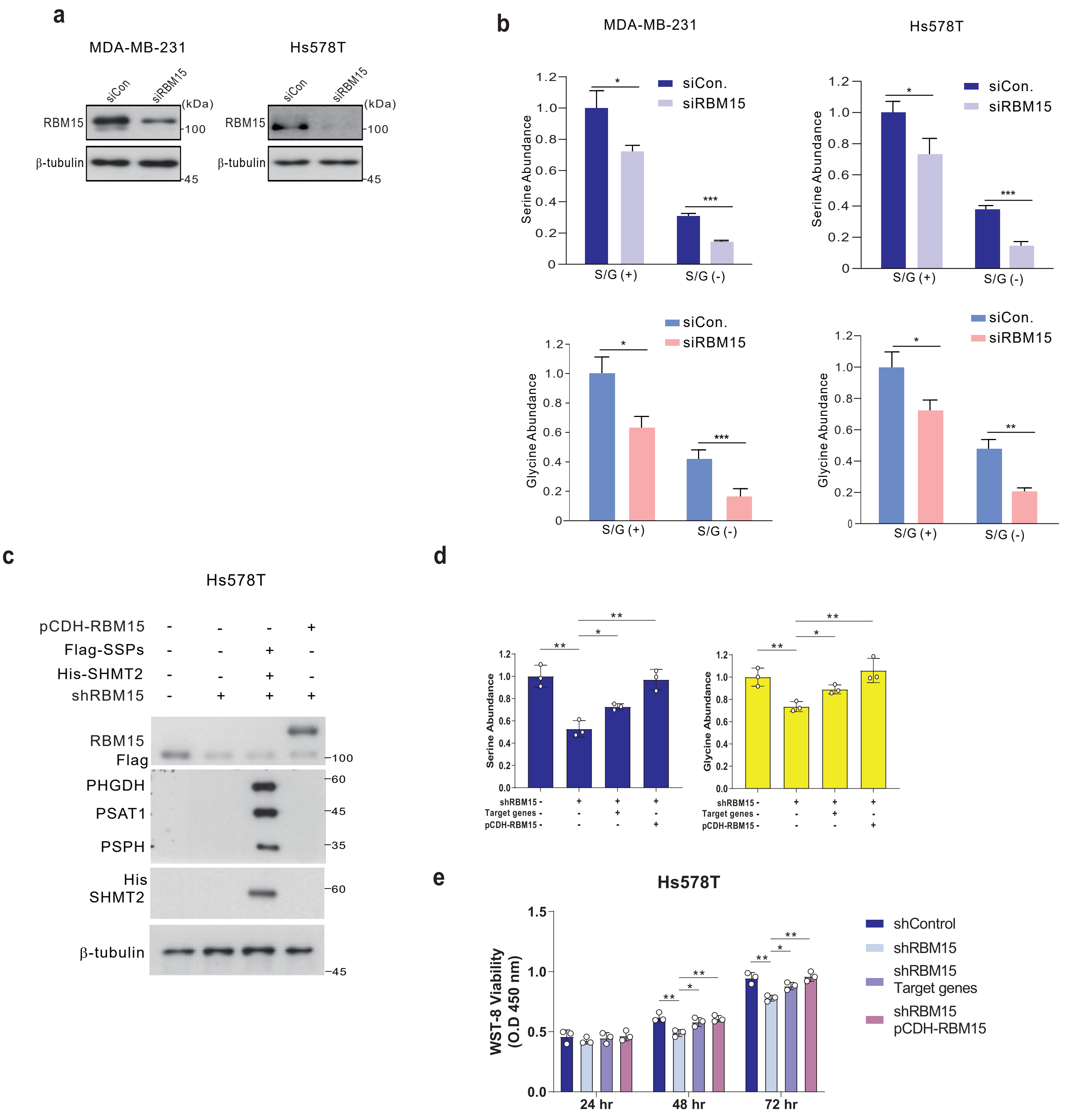

Supplementary Fig. 12| Serine and glycine metabolite change by RBM15 in BC

(a and b), Indicated breast cells were transfected with siRBM15 or siCon. The cells were used for WB with indicated antibodies (a), and for measuring serine and glycine metabolites (b). (c-e), Hs578T cells were transfected with shRNAs and indicated cDNA and used for western blot (c),measuring metabolites (d) and cell proliferation assay (e).Student t-test (two-tailed) was applied to estimate the significance of assay (* *p*<0.05, ** *p*<0.01, and *** *p*<0.005).

Supplementary Fig. 13

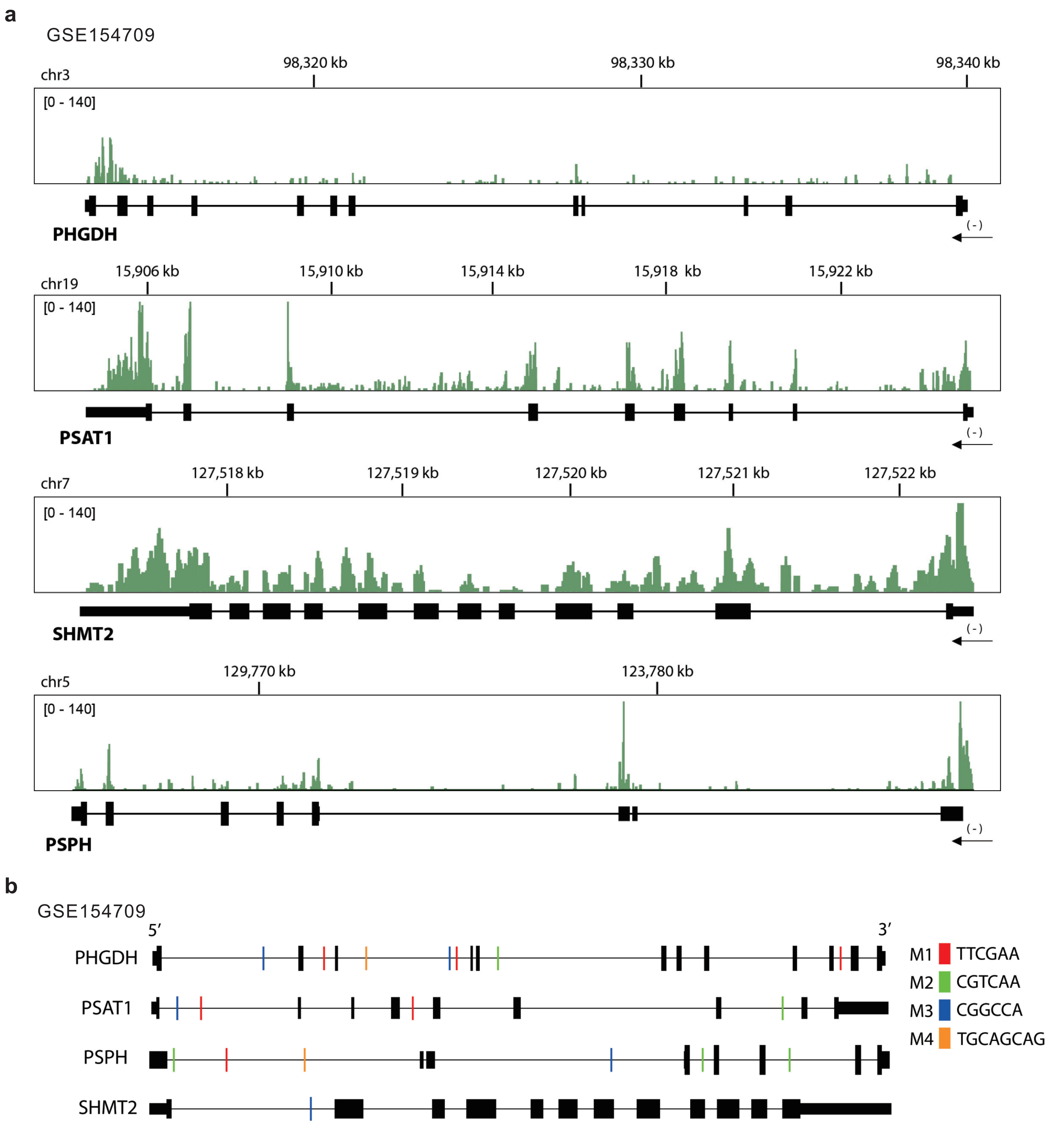

Supplementary Fig. 13| RBM15 binding sites from sequence alignment.

(a and b) Indicated genomic data were analized. UCSC genome browser view of the RIP-seq reads mapping to PHGDH, PSAT1, PSPH, and SHMT2 (a). Sequencing alignment of PHGDH, PSAT1, PSPH, and SHMT2 genomic locus-based on RBM15-binding motif (b).

Supplementary Fig. 14

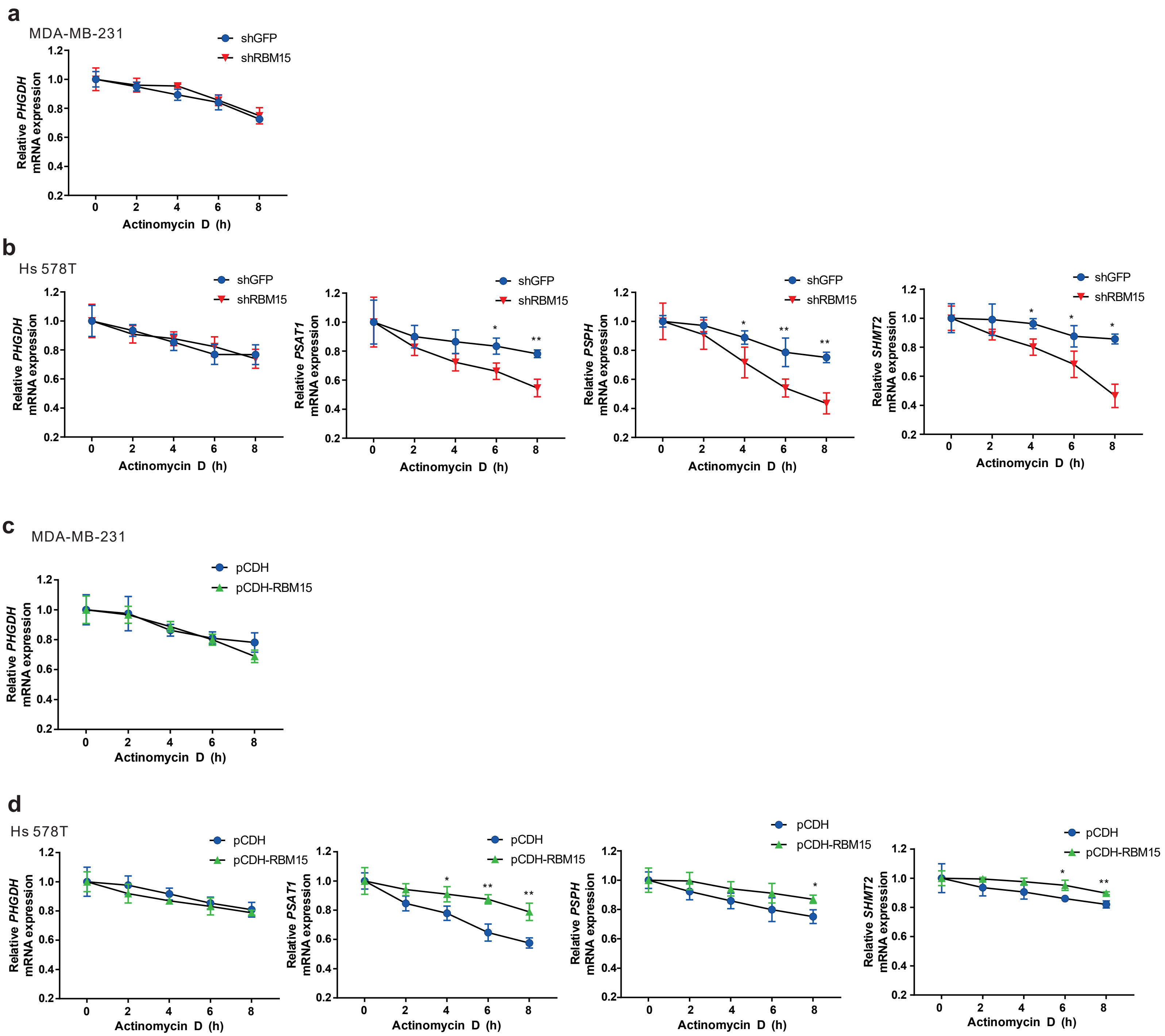

Supplementary Fig. 14| Stability of RBM15 target gene by RBM15 in BC

(a-d) MDA-MB-231 and Hs-578T breast cells were transfected with indicated shRNA (a and b) or cDNA(c and d) and treated with ActD. The cells were harvested at indicated time points and used for qRT-PCR with indicated probe. Student t-test (two-tailed) was applied to estimate the significance of assay (* p<0.05 and ** p<0.01).

Supplementary Fig. 15

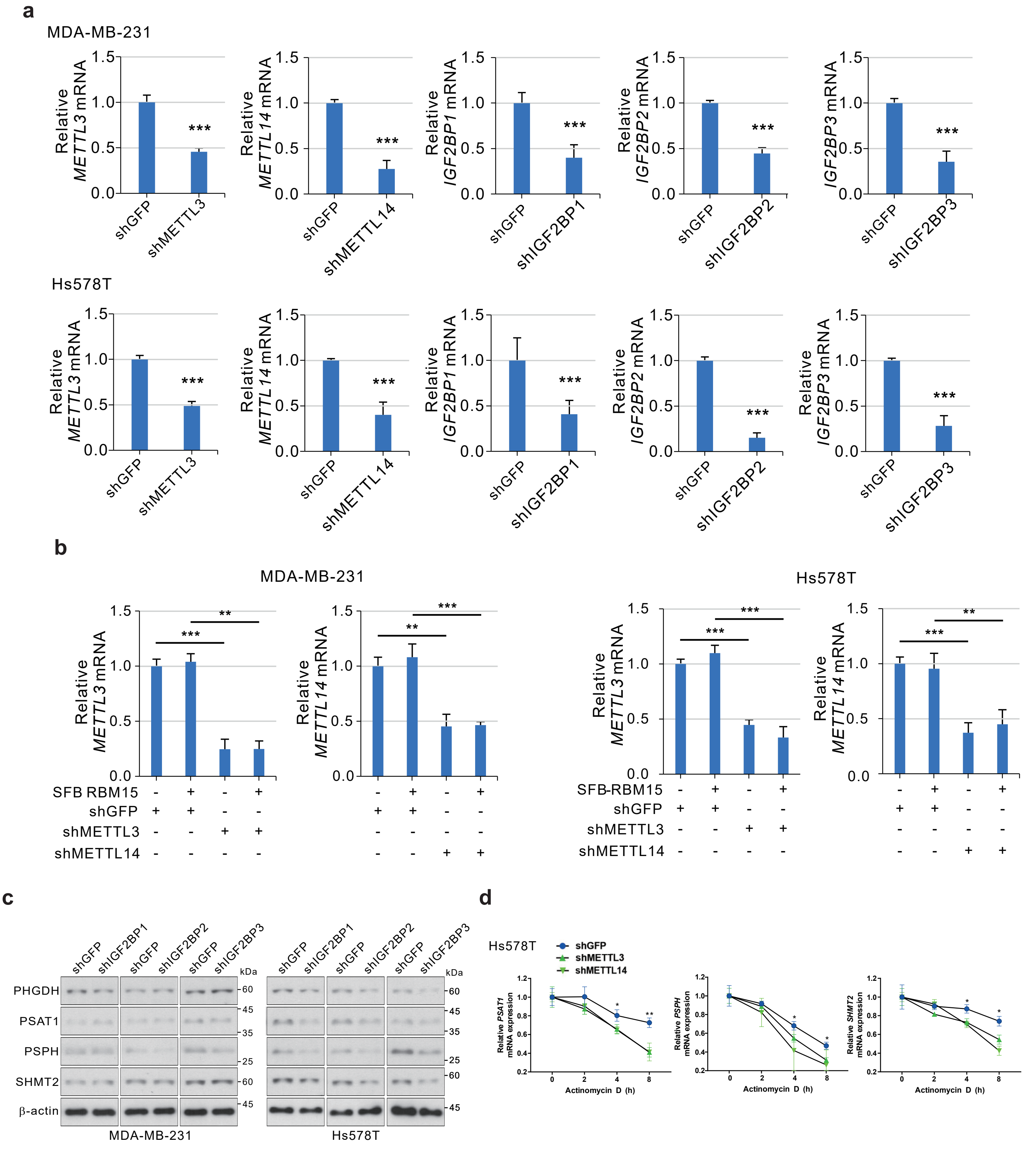

Supplementary Fig. 15| knockdown of m6A writer and reader in BC

(a-c) MDA-MB-231 and Hs-578T breast cells were transfected with indicated shRNAs and cDNA. The cells were harvested and used for qRT-PCR (a and b) and western blot (c). (d) Infected cells were treated with ActD. The cells were harvested at indicated time points and used for qRT-PCR with indicated probe. Student t-test (two-tailed) was applied to estimate the significance of assay (* p<0.05, ** p<0.01, and *** p<0.005).

Supplementary Fig. 16

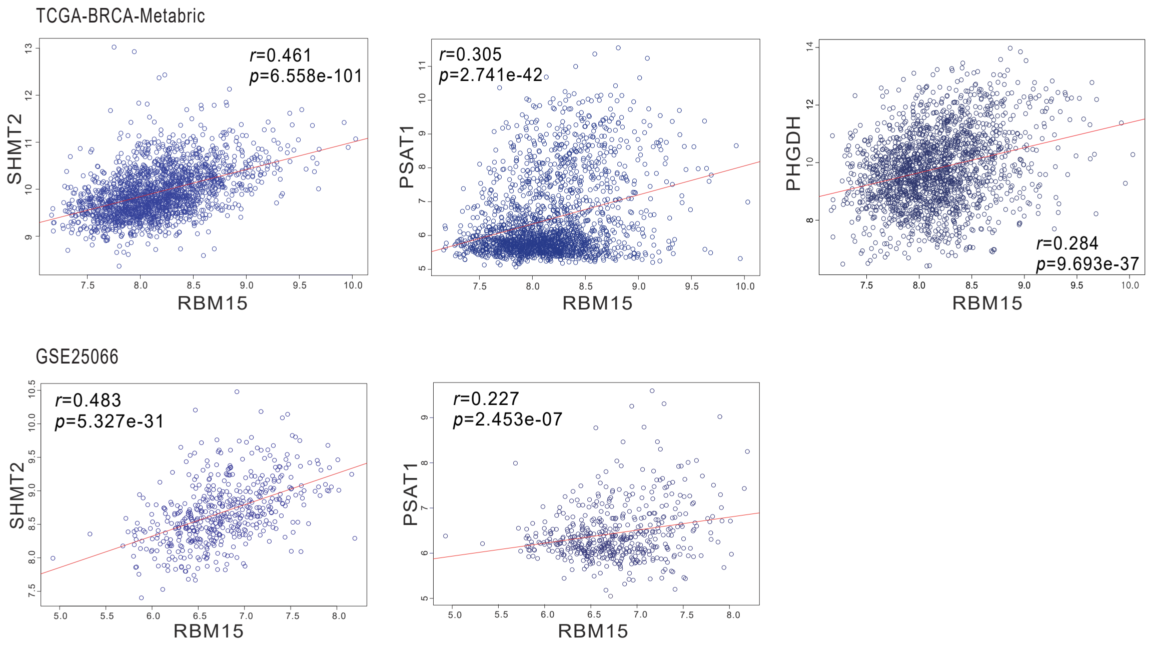

Supplementary Fig. 16| Correlation of RBM15 and its target genes in BC

Correlation scatter plots between RBM15 and PSAT1, SHMT2, and PHGDH in indicated BC cohorts. *p*-value indicates significance of analysis (* $p<0.05$, ** $p<0.01$, *** $p<0.005$, and **** $p<0.001$).

Supplementary Fig. 17

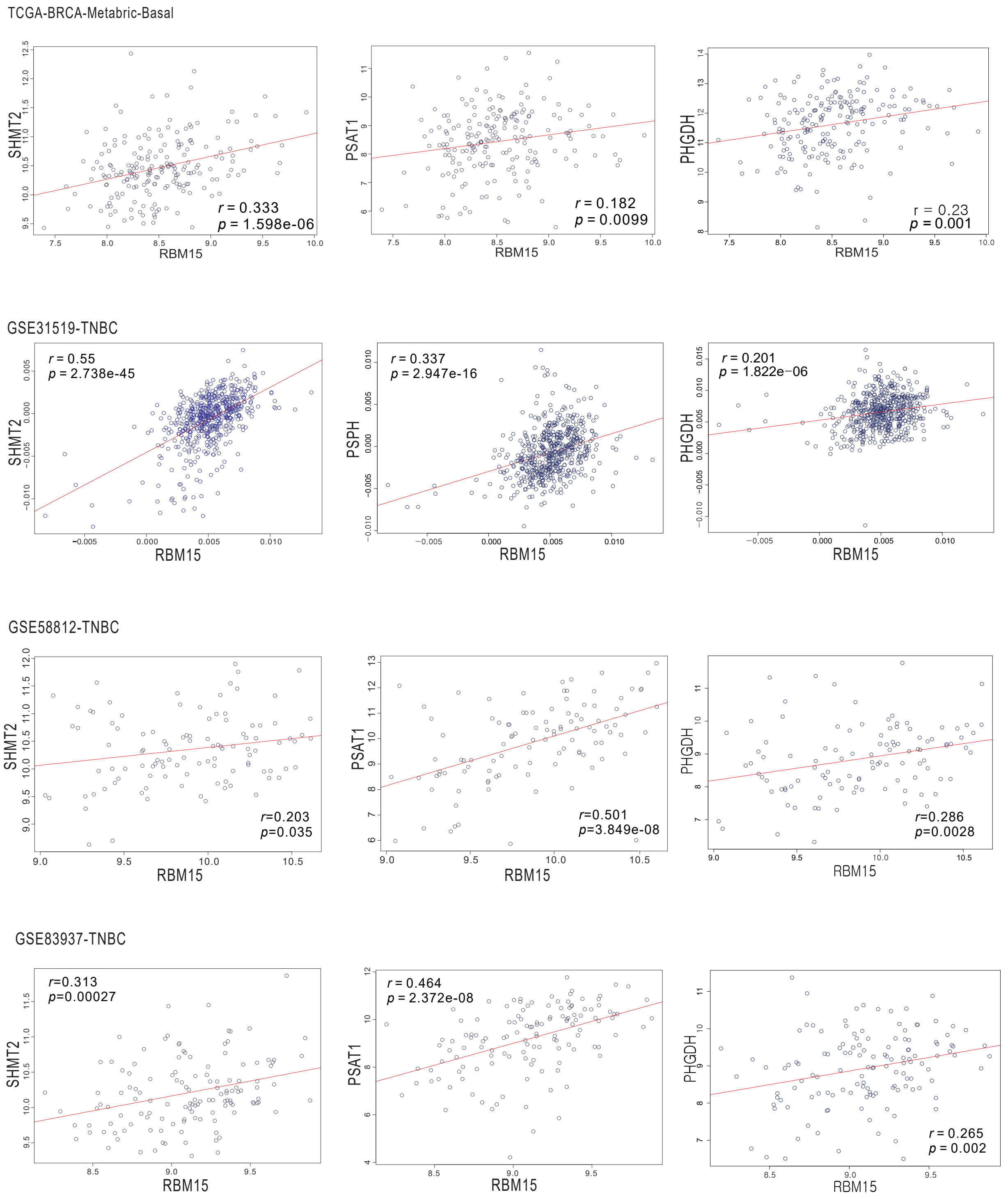

Supplementary Fig. 17| Correlation of RBM15 and serine /glycine metabolism genes in TNBC/Basal BC

Correlation scatter plots between RBM15 and PHGDH, PSAT1, PSPH, and SHMT2 in indicated BC cohorts. *p*-value indicates significance of analysis (* $p<0.05$, ** $p<0.01$, *** $p<0.005$, and **** $p<0.001$).

Supplementary Fig. 18

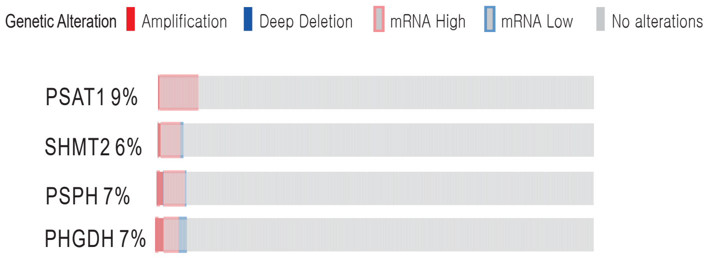

Supplementary Fig. 18| Genetic alteration of serine and glycine metabolism genes in breast cancer.

Genetic alteration of PHGDH, PSAT1, PSPH, and SHMT2 from TCGA-BRCA cohorts. These analysis were done in cBioportal.

Supplementary Fig. 19

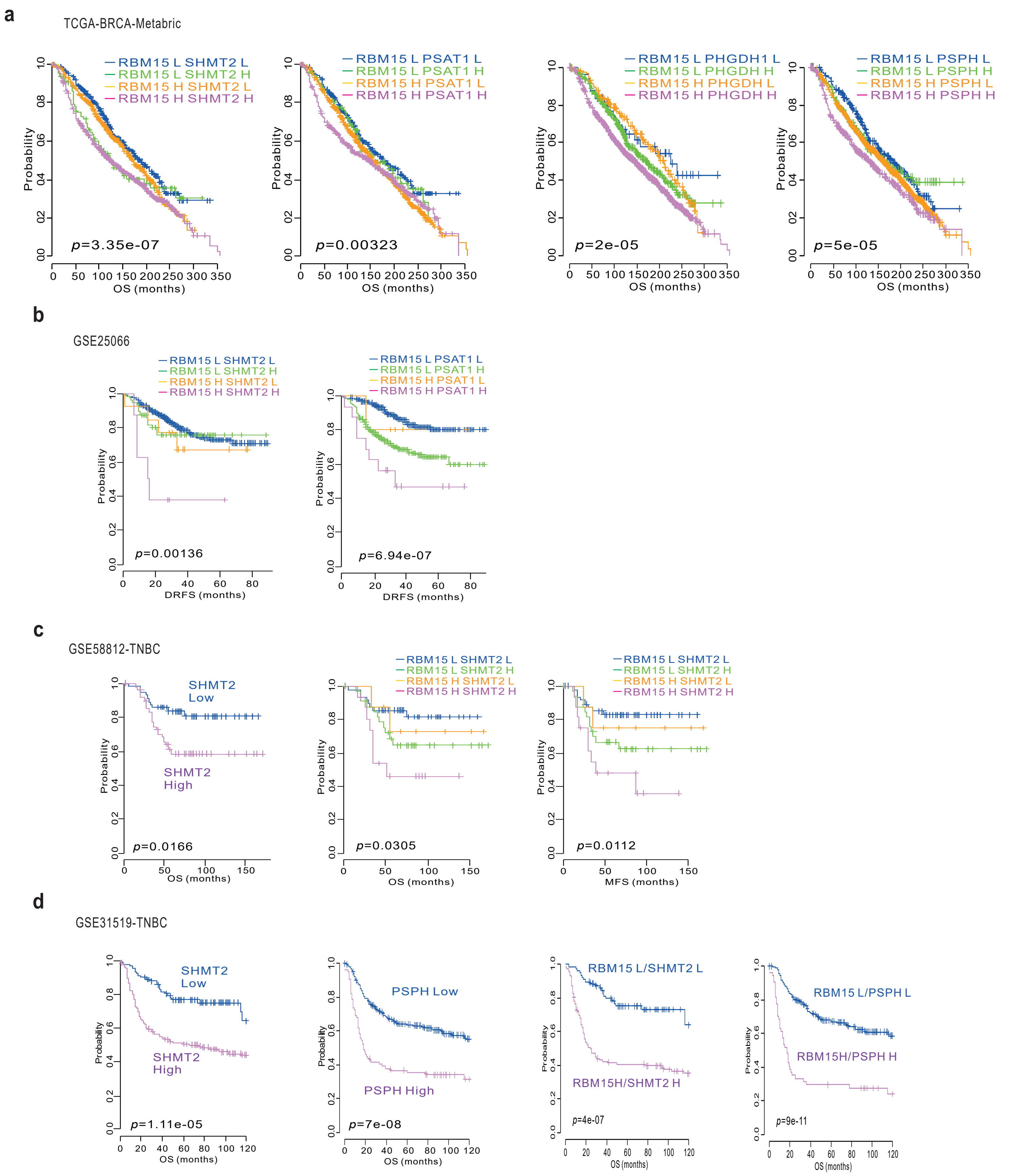

Supplementary Fig. 19| Survival analysis of serine and glycine metabolism genes in BC

(a-d) Using indicated cohort, patients were classified according to the expression level of indicated genes. Kaplan-Meier plots and the log-rank test were used to estimate patient prognosis. *p*-value indicates significance of analysis.

Supplementary Fig. 20

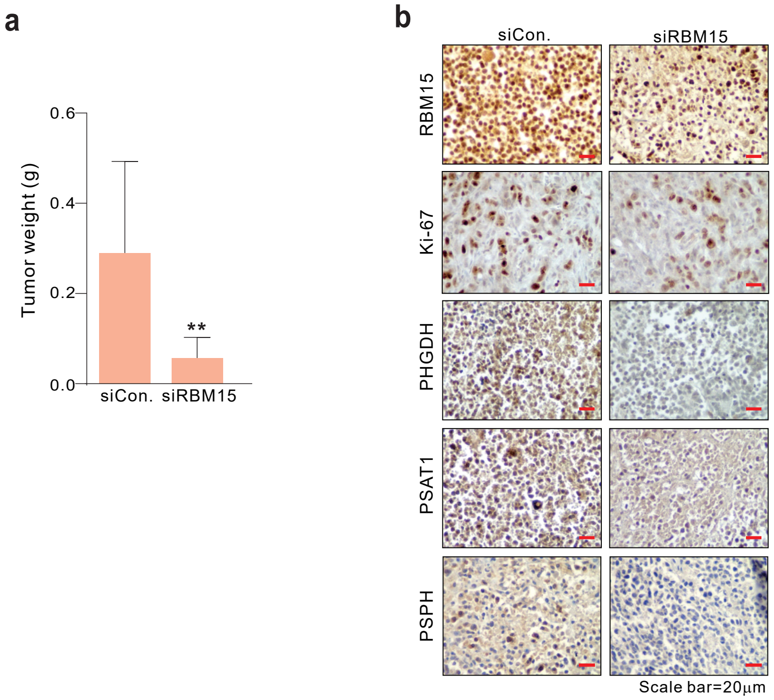

Supplementary Fig. 20| RBM15-silencing reduced SSP gene expression in BC mouse model

(a) After siRNA-containing CH-NP was injected to mice haboring MDA-MB-231 cells, tumor weight was measured (*n* = 10) . (b) The issues were used for IHC with indicated antibody. Representative IHC analysis of mouse samples was performed.
